# Investigating the activity of indigenous microbial communities from Italian depleted gas reservoirs and their possible impact on underground hydrogen storage

**DOI:** 10.3389/fmicb.2024.1392410

**Published:** 2024-04-24

**Authors:** Ruggero Bellini, Nicolò Santi Vasile, Ilaria Bassani, Arianna Vizzarro, Christian Coti, Donatella Barbieri, Matteo Scapolo, Candido Fabrizio Pirri, Francesca Verga, Barbara Menin

**Affiliations:** ^1^Centre for Sustainable Future Technologies, Fondazione Istituto Italiano di Tecnologia, Turin, Italy; ^2^Department of Applied Science and Technology, Politecnico di Torino, Turin, Italy; ^3^Stogit-Snam S.p.A., Crema, Italy; ^4^National Research Council, Institute of Agricultural Biology and Biotechnology (CNR-IBBA), Milan, Italy

**Keywords:** underground gas storage, underground hydrogen storage, underground energy systems, underground methanation, microbial risk/monitoring

## Abstract

H_2_ produced from renewable energies will play a central role in both greenhouse gas reduction and decarbonization by 2050. Nonetheless, to improve H_2_ diffusion and utilization as a fuel, large storage capacity systems are needed. Underground storage of natural gas in depleted reservoirs, aquifers and salt caverns is a well-established technology. However, new challenges arise when it comes to storing hydrogen due to the occurrence and activity of indigenous microbial populations in deep geological formations. In a previous study, four Italian natural gas reservoirs were characterized both from a hydro-chemical and microbiological point of view, and predictive functional analyses were carried out with the perspective of underground hydrogen storage (UHS). In the present work, formation waters from the same reservoirs were used as inoculant during batch cultivation tests to characterize microbial activity and its effects on different gas mixtures. Results evidence a predominant acidogenic/acetogenic activity, whilst methanogenic and sulfate reducing activity were only marginal for all tested inoculants. Furthermore, the microbial activation of tested samples is strongly influenced by nutrient availability. Obtained results were fitted and screened in a computational model which would allow deep insights in the study of microbial activity in the context of UHS.

## 1 Introduction

**Figure 1:**
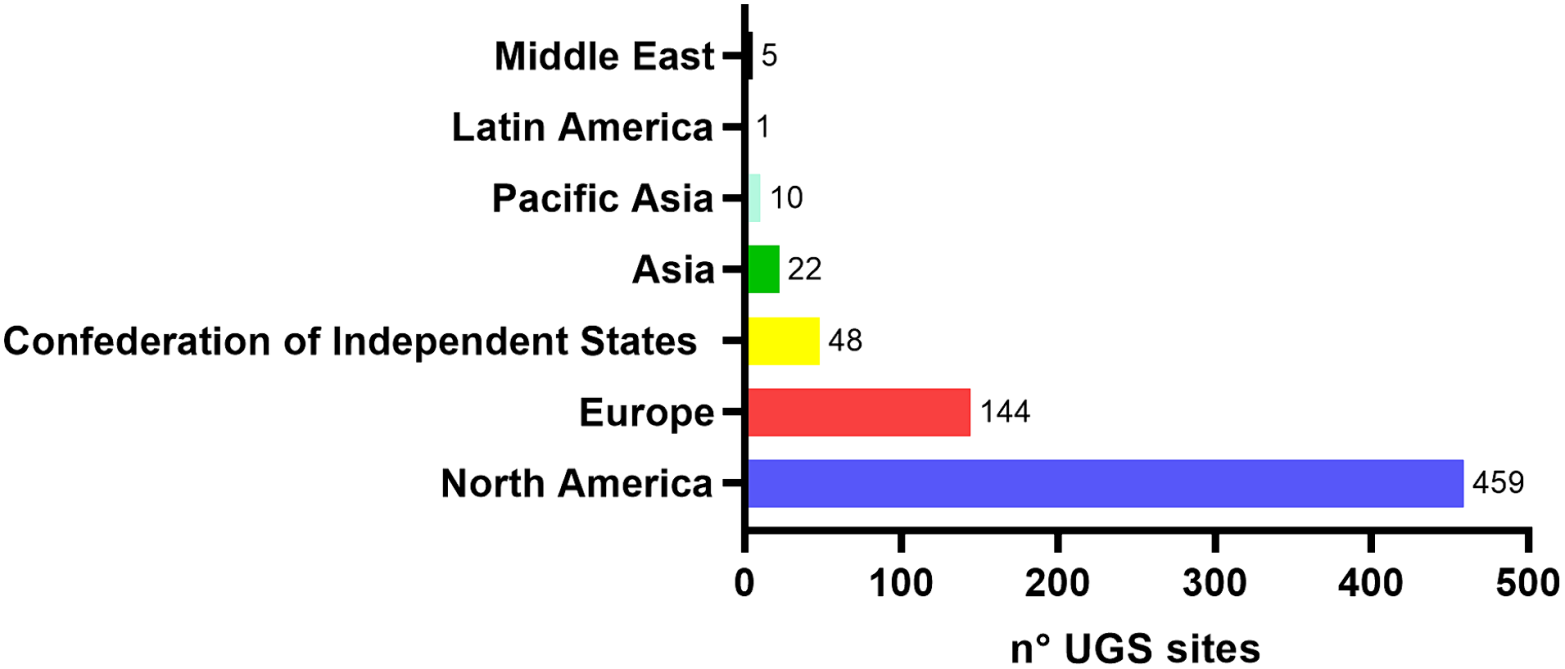
N° of UGS sites and their distribution at global level (adapted from IGU, 2018; Molíková et al., 2022).

In September 2020, the European Commission proposed to further increase both the reduction of greenhouse gases (GHG) emission to 55% and the share of renewable energies from 20 to 32% by 2030. Moreover, long term plans to achieve a carbon-neutral economy by 2050 are being evaluated (European Commission, 2019). Within this context, electricity produced from carbon neutral sources will have to cover the largest portion of future energy demand with its share requested to grow from the current 20 to 60% by the end of the century. However, solar and wind energy are fluctuating, intermittent and need to be balanced to match supply, demand and to ensure electric grid stability. Thus, long-term and large storage capacity of electricity is increasingly required (Götz et al., 2016; Benetatos et al., 2021). To satisfy such needs, Power-To-X (p2X) technologies have been developed with the aim to store energy in the form of different chemical compounds, biofuels and polymers, with the production of H_2_ from the excess of renewables being currently the most investigated and promising Power-To-Gas (p2G) technology (Nazir et al., 2020). When storing energy in the form of synthetic natural gas (SNG) and H_2_, underground storage offers both larger storage capacity and a longer withdrawal period, thus representing an appealing option for long-term energy storage and energy security (Molíková et al., 2022). Leveraging on the long experience of underground storage of natural gas (UGS) in depleted oil and gas reservoirs (Verga, 2018), aquifers, and salt caverns, with a total of nearly 700 UGS facilities worldwide (Figure 1), several recent studies have identified underground H_2_ storage (UHS) as a cost effective technology that would allow peak-shaving capacity in proximity to consumers (Azin et al., 2008; Wang et al., 2013; Zhang et al., 2017; Thorpe et al., 2020). Currently, mainly depleted gas reservoirs and artificial salt caverns are being investigated for UHS (Lord, 2009; Zivar et al., 2021).

Despite the occurrence in deep geological formations of extreme conditions for life (i.e., high pressure and temperature, high salt concentration, potentially high acidity), different studies reported the presence of hydrogenotrophic methanogens (HM), sulfate reducing bacteria (SRB) and acetogenic bacteria (AB) capable of thriving in these sites converted to UGS (Martini et al., 2005; Mochimaru et al., 2007; Morozova et al., 2011; Davis et al., 2012). Due to their metabolisms (Table 1) such microorganisms are of interest when considering the storage of gas blends containing H_2_ as the latter represents the main reducing power required by microbes, and its consumption might lead to the production of new chemical species (i.e., organic acids, alcohols, H_2_S, extracellular poly-saccharides). Microbiological reactions between CO_2_ or H_2_ and the reservoir rock and water, particularly in hydrocarbon reservoirs and in aquifers, can lead to adverse effects (Dopffel et al., 2021; Liu et al., 2023), including: (i) risk to operational safety and deterioration in quality of the stored gas by CH_4_ or H_2_S formation due to the activity of hydrogenotrophic microorganisms consuming injected H_2_; (ii) biocorrosion of technical equipment and pipe clogging through precipitates and biomass accumulation on different infrastructures; (iii) *clogging of the rock pores* due to the growth of microbial biomass causing a reduction of the total storage volume.

**Table 1:** List of microbial metabolisms that could affect UGS including sulfate reducing and methanogenic reactions (adapted from Muyzer and Stams, 2008).

Reaction equation	ΔGoʹ(kJ/reaction)
Sulfate-reducing reactions	
4 H_2_ + SO_4_^2−^ + H+ → HS^−^ + 4 H_2_O	−151.9
Acetate^−^ + SO_4_^2−^ → 2 HCO^−^ + HS^−^	−47.6
Propionate^−^ + 0.75 SO_4_^2−^ → Acetate^−^ + HCO_3_^−^ + 0.75 HS^−^ + 0.25 H^+^	−37.7
Butyrate^−^ + 0.5 SO_4_^2−^ → 2 Acetate^−^ + 0.5 HS^−^ + 0.5 H^+^	−27.8
Lactate^−^ + 0.5 SO_4_^2−^ → Acetate^−^ + HCO_3_^−^ + 0.5 HS^−^	−80.2
Acetogenic reactions	
Glucose → 3 Acetate^−^ + 3H^+^	−310.9
Propionate^−^ + 3 H_2_O → Acetate^−^ + HCO_3_^−^ + H^+^ + 3 H_2_	+76.1
Butyrate^−^ + 2 H_2_O → 2 Acetate^−^ + H^+^ + 2 H_2_	+48.3
Lactate^−^ + 2 H_2_O → Acetate^−^ + HCO_3_^−^ + H^+^ + 2 H_2_	−4.2
Methanogenic reactions	
Acetate^−^ + H_2_O → CH_4_ + HCO_3_^−^	−31.0
4 H_2_ + HCO_3_^−^ + H^+^ → CH_4_ + 3 H_2_O	−135.6
Homoacetogenic reactions	
4 H_2_ + 2 HCO_3_^−^ + H^+^ → Acetate^−^ + 4 H_2_O	−104.6
Lactate^−^ → 1.5 Acetate^−^ + 0.5 H^+^	−56.5

Most of the studies about indigenous microbial consortia from the above mentioned geological formations and available to date in the scientific literature are mainly aimed at the taxonomic characterization based on16S rRNA gene sequencing, whilst only a little information is available about the activity of the microbial consortia when cultivated (Fry et al., 1997; Mochimaru et al., 2007; Basso et al., 2009; Kimura et al., 2010; Timmers et al., 2018). Nonetheless, cultivation experiment are frequently reported as a useful tool in the determination of microbial activity and possible interaction with the chemo-physical environment (Miranda-tello et al., 2007; Liu et al., 2023).

As of 2023, 15 UGS facilities – from the conversion of depleted NG reservoirs - with a total capacity of 19 billion cubic meters are present in Italy (Ministero dell’Ambiente e della Sicurezza Energetica (MASE), 2023).^1^ We have previously characterized formation water samples collected from four depleted NG reservoirs located in different parts of Italy, named R1, R2, R3 and R4, by hydro-chemical and metagenomic analyses (Bassani et al., 2023). 16S amplicon sequencing provided the profile of indigenous microbial populations highlighting the presence of microorganisms (i.e., HM, SRB and AB), which activity might pose a potential issue during future UHS operations. Here, we experimentally assessed the possible risk of activation of the preserved microbial populations from the four previously analyzed reservoirs. We performed batch tests with formation water samples and different gas mixtures, both in the absence and in the presence of nutrient enrichment, carbon sources, mineral nutrients and co-factors (i.e., vitamins, trace elements) shared by target organisms, and monitored the effects of the different microbial consortia on the CH_4_/H_2_ headspace atmosphere. We characterized the growth curves through OD measurement and studied the changes in pH, pressure and gas compositions and the presence of the main metabolic intermediates (e.g., volatile fatty acids). Moreover, using qPCR, the gene copy number variation of functional marker genes linked to the activities of microorganisms, HM (*mcrA* gene), SRB (*dsrB* gene) and AB (*fhs* gene) have been determined. This indicates the activation of the main microbial groups of interest under real and non-limiting nutrient conditions. The data obtained experimentally during 23 days of cultivation were integrated into a biogeochemical model to enable the prediction of the potential microbiological risk during reservoir-scale operations in the long term (90 days).

## 2 Materials and methods

### 2.1 Inocula

The inocula used during the present study was obtained by sampling formation waters in anaerobic conditions from four NG reservoirs which have been previously characterized and named as R1, R2, R3 and R4, as previously described (Bassani et al., 2023). Upon receipt, water samples were stored overnight at 4°C under N_2_ atmosphere to ensure anaerobic conditions. Inoculation procedures were performed in a Whitley A85 anaerobic workstation (Don Whitley Scientific, West Yorkshire, UK) under a steady flow of H_2_, CO_2_ and N_2_.

### 2.2 Batch culture and nutrient media

Batch coltures were performed in serum flasks of a total volume of 158 mL. Flasks were initially closed with rubber septa, flushed with N_2_ to ensure anaerobic conditions and then sterilized in an autoclave (121°C x 80 min). Initially, batch assays were conducted without nutrient enrichment by adding 25 mL of sterilized formation waters to serum flasks seeded with 25 mL formation waters from each reservoir for a total working volume of 50 mL by providing either N_2_, CH_4_ 100% or a CH_4_:H_2_ blend at 50:50 ratio (%/%). Nonetheless, after 25 days no signs of microbial activity were detected (Supplementary Figure S1) and the provision of nutrients was performed in order to stimulate microbial growth. Similarly, in batch assays with nutrient enrichment 25 mL of nutrient media (DSMZ 141) were added to the serum flasks seeded with 25 mL of formation waters from the four reservoirs in order to reach a total working volume of 50 mL. For each reservoir, four conditions have been assessed: (i) one positive control (Glu), i.e., formation water plus DSMZ 141 media sparged with N_2_ and supplemented with 10 mM glucose solution, providing easily accessible carbon source to the microbial consortium and monitoring microbial growth and activity in “ideal” heterotrophic conditions; (ii) one sample with formation water plus DSMZ 141 media supplemented with 100% CH_4_ (CH_4_), mimicking the presence of natural gas within the reservoir; (iii) one sample with formation water plus DSMZ 141 media supplemented with CH_4_/H_2_ mixture at a 90:10 ratio (%/%; H_2_ 10%); (iv) one sample with formation water plus DSMZ 141 media supplemented with CH_4_/H_2_ mixture at a 50:50 ratio (%/%; H_2_ 50%).

For each condition, 3 technical replicates were prepared and supplemented with 2 bar of gas (i.e., CH_4_, CH_4_/H_2_ or N_2_ for samples of the positive control) delivered in the headspace through a sterile gas-tight syringe and a three-way valve. Batches were incubated at 50°C and 200 RPM agitation during a 23-day experiment. Headspace pressure, liquid optical density (OD), and pH were measured using, respectively, a digital manometer, an OD_600nm_ spectrophotometer and a pH meter. Monitoring of the above-mentioned parameters was carried out at 3/4 day intervals.

### 2.3 Volatile fatty acids (VFAs) analysis

To evaluate the concentration of short-chain VFA (scVFA) in the formation waters collected from the four reservoirs and their content after batch cultures, an HPLC methodology developed for the detection of acetic, propionic, n-butyric, iso-butyric, n-valeric, and iso-valeric acid adapted from Ricci et al. (2021) was applied in the present study. Quantification of organic acids was performed by HPLC using a Thermo-Fischer Dionex Ultimate 3,000 system (Thermo-Fischer, USA) coupled with a Thermo-Fischer Dionex Ultimate 3,000 Variable wavelength detector operating at 210 nm. For organic acids, the column (Metab AAC, ISERA GmbH, Düren, Germany) was eluted isocratically with 9 mM H_2_SO_4_ at a flow rate of 0.6 mL min-1 and an oven temperature of 40°C. Calibration was performed by generating individual stock solutions (100 mM) of the above-mentioned scVFA species by solubilizing their relative weight/volume in MilliQ water. Stock solutions were then used to prepare calibration standards containing all the acid species for concentrations between 10 and 1 mM. Data collected were screened against the VFA levels contained in the media and in the inactivated formation waters used as blanks allowing to differentiate between the degradation/generation of VFAs naturally occurring in the culture media from that caused by microbial activity.

### 2.4 Gas chromatography analysis

Headspace gas concentrations were measured using gas chromatography (MicroGC Fusion, INFICON, Bad Ragaz, Switzerland) calibrated for the detection of H_2_, O_2_, N_2_, CH_4_, CO_2_ and H_2_S (%). 3 mL headspace gases were sampled at cultures inoculation (Day 0) and at the end of the cultivation cycle (Day 23) using a gas-tight luer lock syringe. Data displayed in this study are obtained from the average of single measurements performed on physiological triplicates for each condition tested and have a max RSD <0.5%.

### 2.5 qPCR methodologies for the quantification of HM, SRB and AB

To evaluate abundances of HM, SRB and AB during batch cultivation of formation waters collected from R1, R2, R3 and R4 the qPCR protocol described by Nyyssönen et al. (2012) targeting genomic sequences of both *mcrA* and *dsrB* and relying on the use of primer couples ME1/ME3R and DSRp2060F/DSR4R was adopted. Similarly to HM and SRB, AB abundance was assessed by qPCR targeted the fhs gene encoding for formyl-tetrahydrofolate ligase, using the FHS 2 and FTHFSr primers previously described by Xu et al. (2009). The protocol consists of a Sybr-green qPCR assay using the Quantitect Sybr Green Kit (Qiagen, Hilden, Germany), single reactions volume was 25 μL, with all primer couples added at a final concentration of 0.3 μM and with 2.5 μL genomic DNA template. The assays were carried out with a Qiagen Rotor-Q thermal cycler (Qiagen, Hilden, Germany), fluorescence was detected within the range of green (510 nm) in a final 25 μL reaction volume. Amplification conditions were as follows:

Initial denaturation: 15 min 95°C;

Denaturation: 40 s 95°C;

Annealing: 40 s 55°C;

Elongation: 40 s 72°C;

Final elongation: 6 min 72°C.

qPCR specificity was tested through melting curve analysis consisting of an initial denaturation for 10 s at 95°C followed by re-annealing at 55 degrees with a gradual T increase up to 95°C while fluorescence was continuously detected. The lowest n°copies/ml detectable by the used methodology were 1.33 × 10^2^, 1.85 × 10^2^ and 1.76 × 10^2^ copies/ml for *mcrA*, *dsrB* and *fhs*, respectively. Samples with values below this threshold were not reported.

Results obtained were statistically analyzed by means of a one-way ANOVA test. The results reported in the present work are those with *p* < 0.03. Results of statistical analysis on significant samples are described in the Supplementary Figures S2–S4.

### 2.6 Computational model description

A 0-D model was developed using the COMSOL® Multiphysics platform, a finite element modeling program designed to solve a vast array of partial differential equations (PDEs), which has emerged as an innovative and succesfull approach to groundwater modeling (Li et al., 2009; Jin et al., 2011; Jin and Kirk, 2016; Liu and Liu, 2017; Sainz-Garcia et al., 2017).

The developed model allows determine the growth kinetics and gas/VFA production and consumption, accounting for the concentration in moles of target chemicals and their presence in both gas and liquid phases.

Governing equations for the four main microbial processes, represented by reaction equations outlined in Supplementary Table S1, are implemented in the model simulations (Hoehler et al., 1998; Conrad, 1999; Jin and Bethke, 2005; Feldmann et al., 2016; Jin and Kirk, 2016; Berta et al., 2018; Delattre et al., 2020; Strobel et al., 2023; Tremosa et al., 2023).

The model takes into account/includes equilibrium reactions for gas-water interactions, focusing on potential increases in dissolved carbonate and sulfate in reservoir water for bacteria and archaea reactions. Equilibrium calculations are based on the mass action law, considering all species used in this study and their corresponding equilibrium constants. Supplementary Table S1 details the equilibrium phases, mass action equations and constants (Hemme and van Berk, 2018; Louca et al., 2019).

Monod model is a standard in modeling microbial growth under substrate limited conditions. Due to the dependence of microbial growth and decay on substrate and electron acceptor availability the “double Monod model” has been proposed to address this (Megee et al., 1972; Strobel et al., 2023), as outlined in the Supplementary Table S1.

The model accounts for changes in key components across both gas and liquid phases. The concentration of components within the liquid phase, or solubility, is generally represented using Henry’s Law. A crucial assumption here is the equilibrium between concentrations in both phases.

The mathematical model is based on the two-film theory and the “film” between the phase is the resistance against the mass transfer. As no reaction takes place in the gas phase, the change of the substrates hydrogen and carbon dioxide over time is dominated by the mass transfer (Li, 2017). With a mass-transfer model emulating molecular flow between water and gas phases, by combining mass transfer with microbial reactions, we can derive equations for the production and consumption of H_2_, H_2_S, CO_2_, and CH_4_ within the liquid phase. Mass transport is described using a generalized approach (Hagemann et al., 2016) on the COMSOL^®^ multi-physics platform, as presented in the Supplementary Table S1. To effectively address mass balances, fluid dynamics, and kinetic equations the following boundary conditions and model specific assumptions were made:

*Boundary Conditions*:

■ Mass transfer is considered between the liquid culture and fed gas.
■ In a Batch configuration, given that there’s no liquid exchange, velocities in the inlet/outlet areas are zero.
■ Model parameters include temperature, inlet gas quantity, volume fractions, initial pressures, and microbial and nutrient types and initial amount.
■ We incorporated operational data from experiments to input initial and working parameters into the models.
■ Metagenomics analysis (Bassani et al., 2023) and low-pressure batch tests with formation water informed initial microbial compositions and active species.
■ Biochemical kinetics for main metabolisms in the reservoir were derived from experimental data.

*Model Assumptions*:

■ Only active microorganisms in batch tests with formation waters of the four reservoirs were considered.
■ For study cases involving the addition of all essential nutrients in the liquid phase, we considered only the initial addition as outlined in section 2.3.
■ We used results from the hydro-chemical analysis of formation waters for each reservoir to inform the model about bicarbonate, sulfate, organic carbon amounts, and pH values.
■ Equilibrium reactions were simulated to account for possible dissolved carbonate/sulfate presence in the formation water for archaea and bacteria biochemical reactions.
■ The liquid phase is saturated with N_2_, CH_4_, and H_2_, varying based on the study case. The initial amounts of N_2_, CH_4_, and H_2_ match the solubility limit for each gas at operating pressures (Duan et al., 1992; De Lucia et al., 2015; Reitenbach et al., 2015).
■ CO_2_ is considered for both liquid and gas phases.
■ The model considers the influence of operational conditions on bacterial growth rates

### 2.7 Model validation

The validation of the model involves verifying its operational efficacy through comparison between simulated and experimental data, under identical operating conditions, utilizing the COMSOL® platform. A model-fit procedure is adopted, wherein the values of unknown parameters, namely the biochemical kinetics constants, are adjusted to minimize the discrepancy between model predictions and available experimental data. A specific comparative analysis between the simulated and experimental data is conducted via statistical error analysis. This method implements a constrained search procedure, thus necessitating the specification of lower and upper parameter limits. These limits were selected based on the range of values most commonly observed in relevant literature. As delineated in the preceding section, comprehensive knowledge on mass transfer and bacterial growth kinetics has been wholly integrated into formulating the modeling equations, with the resultant model being implemented within the COMSOL® computing platform. To derive suitable estimates for the parameters vital for mathematical representation, reliance is placed on either experimentally determined values or an inference procedure fitting model simulations to observed data. In particular, comparisons between simulated and experimental data are typically executed using a statistical error analysis based on the Levenberg–Marquardt method, complemented with the second least-squares regression technique to minimize the residual (Vasile et al., 2021). The exploration of parameter space ceases when the model simulation optimally aligns with the experimental data.

In the initial phase of the modeling work, the model will be utilized to verify its operational efficacy by scrutinizing the concordance between the simulation estimates and the experimental measurements for growth rate, VFA, methane, carbon dioxide, hydrogen sulfide and hydrogen evolution derived from lab tests. The conditions listed in Table 2 were simulated along all the modeling campaign. The model operation were scrutinized by comparing them with the experimental data reported in paragraph 3.4 and in the Supplementary Information. The parameters pertaining to biochemical kinetics, gas–liquid equilibria, and bacterial growth were adjusted using the experimental data. Furthermore, a comparison was made between the results obtained from the simulations and the experimental data in terms of the number of HM-AB-SRB cells, headspace gas pressure and composition, and VFA produced.

**Table 2:** Operative conditions simulated with the biochemical model.

Total volume (ml)	158
Gas volume (ml)	108
Liquid volume (ml)	50
Nutrient liquid volume (ml)	25
Formation water (ml)	25
Headspace pressure (bar, g)	2
Reservoirs simulated	4
Simulated tests for each reservoir	4
1 test	10 mM glucose
2 test	100% CH4
3 test	90CH4-10H2
4 test	50CH4-50H2
Time for each test (days)	23–90
Temperature – K	323
HCO_3_ in nutrient (g/L)	5

## 3 Results

### 3.1 Evolution of OD and pressure trends during batch culture tests

During the experiments, the cultures O.D. and pressure variations were monitored to evaluate the microbial growth and activation. When cultured in the absence of nutrients, the formation waters of the four reservoirs did not display any sign of microbial growth. O.D. and pressure measurements did not reveal significant changes when compared to negative controls thus underlying limiting conditions for microbial growth (Supplementary Figure S1).

**Figure 2:**
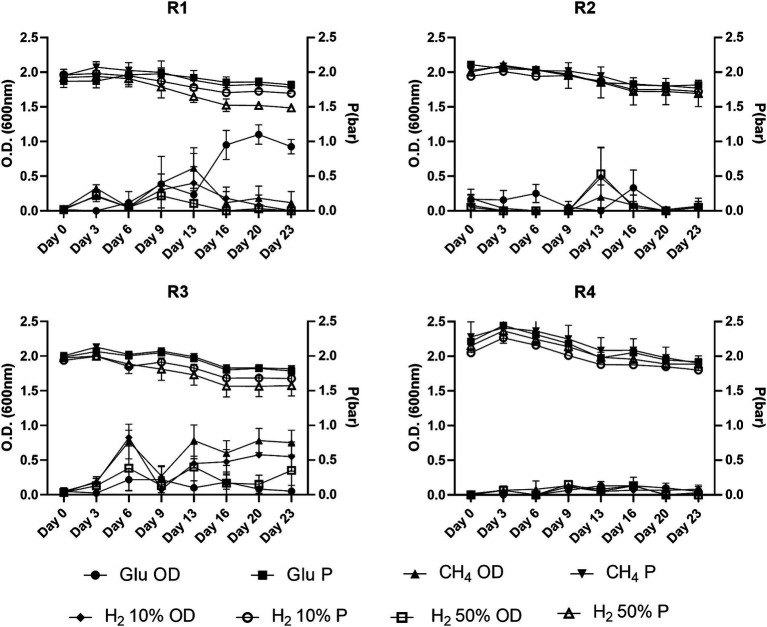
O.D. and P variations observed during batch cultivations of formations water of the four reservoirs object of the present study.

Data related to O.D. and pressure values for the batch tests performed in the presence of nutrients are reported in Figure 2. The batches prepared from the R1 formation water supplemented with gas mixtures reached O.D. 0.5 within the first 9 days, whilst in glucose-fed batches further increase was detected until day 20. Batches of formation water from R3 glucose-supplemented samples did not exhibit increases in the O.D. values which were reported below 0.2. Nonetheless, R3 supplemented with gas showed remarkable O.D. increase rates within the first 13 days with values ranging from 0.6 to 1 in batch cultures with headspace filled with 50% H_2_ and CH_4_, respectively, (Figure 2). Conversely, from what we observed in cultures seeded with R1 and R3 formation waters, the inoculant collected from reservoirs R2 and R4 did not show significant variations of the O.D. during the experiment, with values remaining frequently <0.3 (Figure 2).

Along with the O.D. variations, the headspace partial pressure was monitored during the whole experiment and the data are illustrated in Figure 2. A common trend was observed in most of the tested conditions, with the average headspace pressure reported to decrease ≈ by 0.1–0.3 bars by the end of the experiment. Nonetheless, with pressure losses observed in positive control samples (ΔP ≤0.3 bar) falling within the same range of those reported for most of the batch cultures, the observed variations are attributed to the numerous samplings performed during the batch experiment. Only the batch cultures from R1 and R3 with 50% H_2_ in the headspace displayed ΔP higher than the positive control ones, with ΔP_R1H250%_ = 0.13 bar and ΔP_R3H250%_ = 0.1 bar, respectively.

### 3.2 VFAs analysis and pH

**Figure 3:**
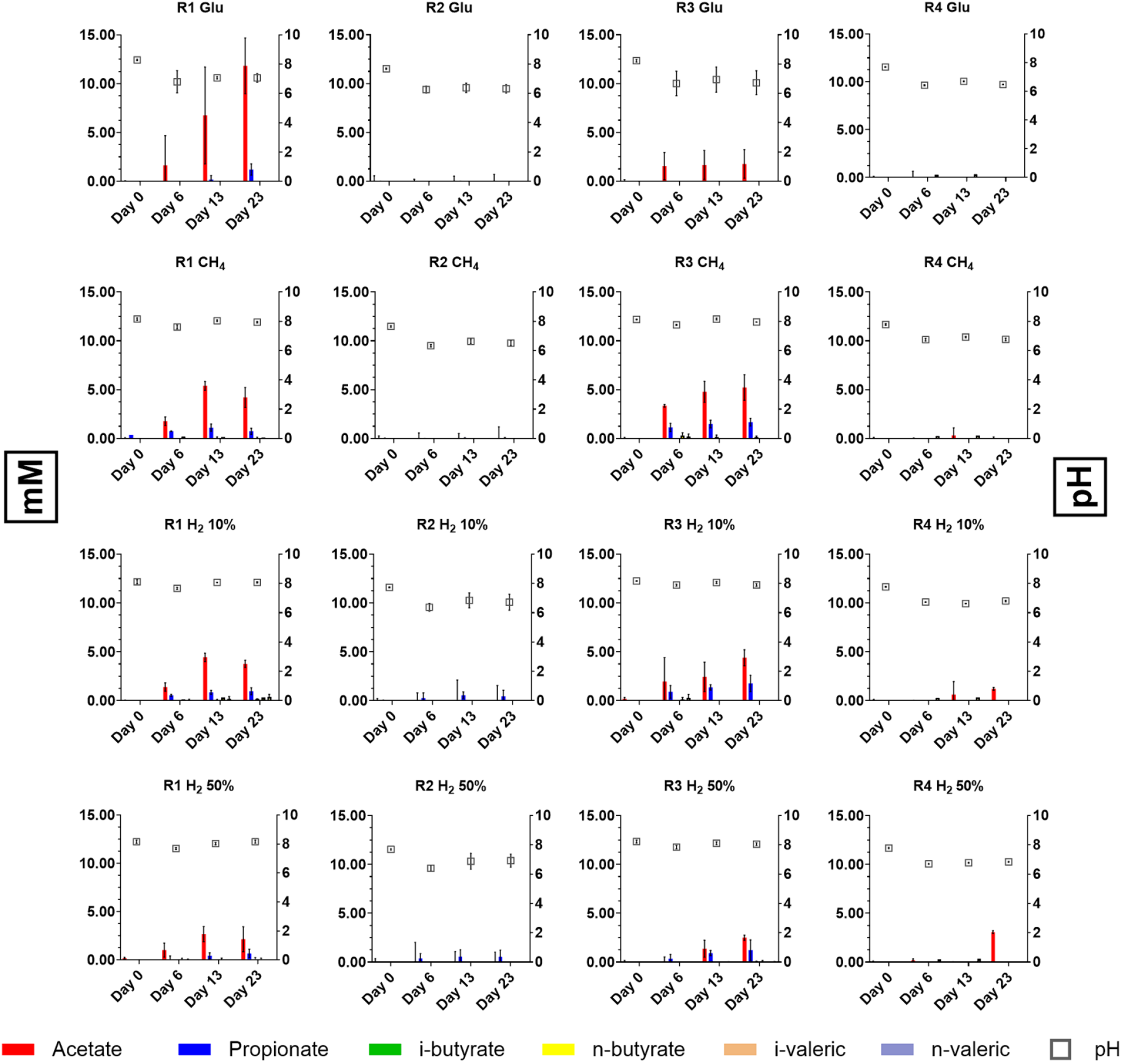
Differences in scVFA concentrations and dynamic reported during batch cultivation of formation waters from reservoirs R1, R2, R3, and R4.

Given the importance that VFAs have as metabolic intermediates in microbial metabolism, their quantification and monitoring could elucidate interactions occurring within the mixed microbial communities of the reservoirs. The presence and quantification of five species of VFAs, namely acetic, propionic, n-butyric/iso-butyric and n-valeric/iso-valeric acid, were monitored using HPLC during batch experiment, results of the analysis are resumed in Figure 3.

At the time of the experiment, it was observed that in samples from both R1 and R3, variations in the concentrations of VFAs occurred in all conditions tested. Acetate and propionate were the most represented VFAs species although within different concentration ranges (Figure 3). In samples from R1 cultures acetate was reported between 2.13 and 11.81 mM by day 20 with positive controls fed with glucose displaying the highest concentrations (11.81 mM). In samples from R3 acetate was reported between 1.72 and 5.21 mM. For what concerns propionate, concentrations between 0.68–1.21 mM and 0.01–1.21 mM were observed in R1 and R3 samples by day 20, respectively. Along with acetate and propionate, other scVFAs species were detected although in lower and variable amounts. In particular, during the cultivation of formation waters from the four reservoirs traces of i-butyric, butyric and i-valeric acid were detected (Figure 3).

While variations in scVFAs levels occurred in R1 and R3 cultures, data collected from cultures inoculated with R2 and R4 formation liquids evidenced consistent differences with the other reservoirs. Samples collected from cultures supplied with glucose and CH_4_ displayed no sensible variations in levels of considered scVFAs species. R2 samples in which the serum headspace was filled with mixtures of 50% H_2_ and 10% H_2_ traces of propionate, butyrate and i-valerate were reported whilst in R4 samples provided with 50% H_2_ a peak 3.06 mM peak of acetate was detected on day 23 (Figure 3).

Regarding pH, batch cultures obtained from R1 and R3 stabilized their pH between 7.5 and 8 by day 23, whilst cultures obtained from R2 and R4 reached pH between 6.5 and 7. pH values measured during batch cultivation were slightly higher or within the ranges previously reported (Bassani et al., 2023). Nonetheless increase in average pH is likely to be attributed to interactions with the provided media, which could have partially altered the pH. This is also sustained by the fact that in batch culture performed without external nutrient provision pH values remained stable along the experiment (Data not shown) and close to those previously reported by chemical characterization Bassani et al. (2023).

### 3.3 qPCR and GC analysis

To better define the growth of the microbial clusters of interest (i.e., HM, SRB and AB) during batch cultivation of formation waters of R1, 2, 3 and 4, qPCR analysis was performed on samples collected at the beginning of the test on day 0 (Table 3), in the mid-phase at day 13 and at the end at day 23.

**Table 3:** Initial concentrations in copies/ml for target genes enumerating methanogens (*mcrA*), SRB, (*dsrB*) and AB (*fhs*).

Reservoir	*mcrA*		*dsrB*		*fhs*	
	Average	sdev.	Average	sdev.	Average	sdev.
R1	2,42 × 10^3^	1,32 × 10^3^	n.d	n.d	1,09 × 10^4^	5,69 × 10^2^
R2	n.d.	n.d.	n.d	n.d	1,79 × 10^2^	1,25 × 10^2^
R3	5,72× 10^2^	4,21 × 10^1^	n.d	n.d	1,04 × 10^4^	5,94 × 10^2^
R4	n.d	n.d	n.d	n.d	n.d	n.d

**Figure 4:**
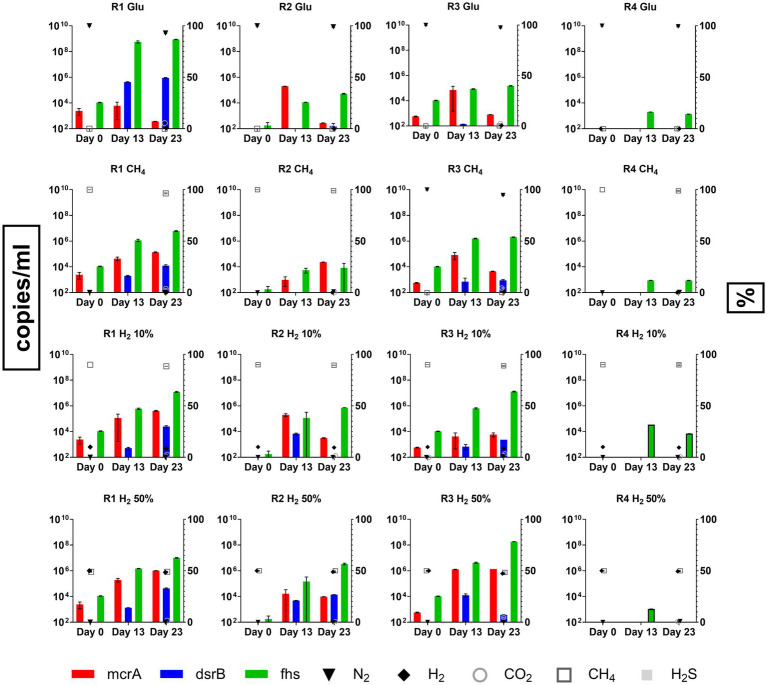
Copies/ml for functional genes from HM (*mcrA*), SRB (*dsrB*) and AB (*fhs*) enumerated using qPCR during batch cultivation of R1, R2, R3 and R4 formation waters under different CH_4_/H_2_ gas mixtures.

qPCR results obtained from the different conditions tested are reported in Figure 4, along with the concentrations of headspace gases measured at the beginning (day 0) and at the end of the experiment (day 23). qPCR analysis on day 0 samples revealed the initial composition of the inoculant obtained from R1, 2, 3 and 4. *Fhs* gene was the most abundant among those used as targets with values of 1.09 × 10^4^, 1.79 × 10^2^ and 1.04 × 10^4^ copies/ml in R1, R2 and R3, respectively. *mcrA* gene was detected in R1 and R3 at 2.42 × 10^3^ and 5.72 × 10^2^ copies/ml respectively, whilst in R2 samples *mcrA* copies/ml were reported below the threshold of detection. *dsrB* copies/ml were the least abundant in all samples and were frequently reported below the threshold of detection of the devised technology. Among the tested reservoir samples from R4 were the only ones that gave no significant response to the assessed gene markers with copies/ml of *mcrA*, *dsrB* and *fhs* being consistently below their relative threshold of detection.

qPCR analysis also revealed that copies/ml of *fhs* gene targeting AB were the most abundant between analyzed samples, with their number increasing significantly (*p* < 0.0001; Supplementary Figures S2, S3) along the incubation reaching by day 23 values between 6.01 × 10^6^–8.7 × 10^8^ copies/ml and 1.53 × 10^5^–1.85 × 10^8^ copies/ml in samples from R1 and R3, respectively. Similarly, *fhs* gene marker was the most abundant in R2 samples cultivated in H2-supplemented cultures. Nonetheless, copies/ml of the target gene were reported at lower amounts (8.4 × 10^3^–3.4 × 10^6^ copies/ml) than both R1 and R3. *mcrA* copies/ml, used for targeting the presence of HM, were measured at several orders of magnitude below (1.33 × 10^2^–1.37 × 10^7^) those of *fhs* in several samples from R1, 2 and 3. Although most of the variations observed for *mcrA* copies/ml were not statistically relevant, significant (*p* < 0.0001) increases in target gene copies abundance was reported in samples from R1 and R3 cultivated under 50% H_2_ headspace with copies/ml on day 23 counting at 1.03 × 10^6^ and 1.37 × 10^6^, respectively (Figure 4). For what concerns SRB copies/ml of the target gene used in the present study, *dsrB*, were often reported below or slightly above the threshold level of the devised methodology (1.85 × 10^2^ copies/ml) in cultures from R2, R3 and R4 (Figure 4). An increase in *dsrB* copies/ml value was observed in R1 samples with control cultures supplemented with glucose stabilizing by day 23 at 9.03 × 10^5^ copies/ml, whilst other R1 cultures reported values between 1.24–4.3 × 10^4^ copies/ml. Further relevant increases in target gene copy numbers were only observed in R2 and R3 samples cultured under H_2_ 10 and 50% headspace with *dsrB* copies/ml stabilizing between 3.75 × 10^2^ and 1.4 × 10^4^ copies/ml. Data regarding headspace gas concentrations in the different conditions tested for the four reservoir waters are plotted along with qPCR data in Figure 4. By the end of the experiment on day 23, all cultures from both R1 and R3 displayed an increase in CO_2_ headspace concentrations with values ranging between 1.5–5.5% and 2.5–4.5%, respectively. Furthermore, both R1 and R3 positive control cultures and those gassed with 100%CH_4_ displayed production of H_2_ in low amounts (0.5–1.5%). In most of the tested conditions, along with the reported increase in CO_2_, a proportional decrease in the concentrations of either N_2_, CH_4_ or CH_4_/H_2_ mixture occurred. No traces of CH_4_ were detected in positive control samples, whilst its measurement in other samples was covered by the CH_4_ already present in the headspace atmosphere. Finally, traces of H_2_S were detected only within R1 positive controls and cultures supplemented with H_2_ 10% at values in the range between 0.045 and 0.05%, whilst no traces of the gas were reported in other cultures where SRB growth was detected.

With respect to the cultures from R2 and R4, the monitoring of the headspace gas concentrations did not reveal significant changes in any of the tested conditions.

### 3.4 Computational modeling results

**Figure 5:**
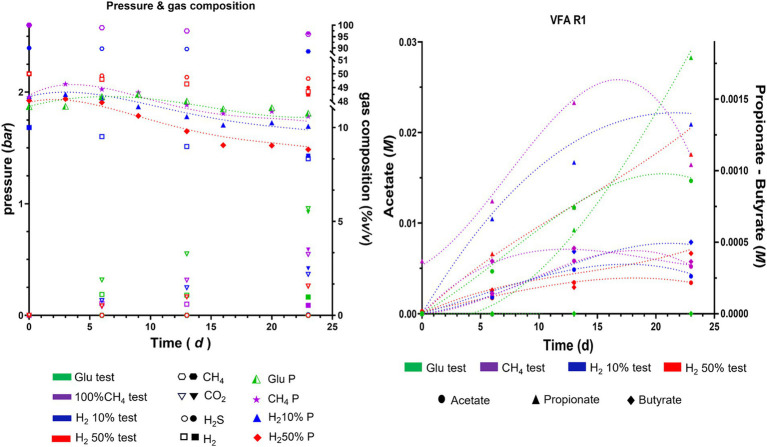
Experimental (filled symbol) and simulated trends (dash lines for pressure and VFA, empty symbols for gas phase) of pressure, gas composition and VFA during batch cultivation of R1 formation waters under different CH_4_/H_2_ gas mixtures.

A modeling procedure was used to simulate microbial activity in the four reservoirs of interest for the case studies analyzed. Due to the effects that indigenous microbial communities could have on the stored gases, variations of headspace pressure and gas concentrations/partial pressures (i.e., CH_4_, H_2_ CO_2_ and H_2_S) were considered along with possible pressure drops due to the liquid and gas sampling. The results of the simulations on headspace gas compositions, pressures and VFA production are shown in Figure 5 (R1) and Supplementary Figures S6, S7 (R2, R3 and R4) in comparison to data obtained from experimental trials with external nutrient provisions. In these figures, each color represented in the legend corresponds to a specific case study: green for glucose tests, magenta for 100% CH4, blue for 10% H2, and red for 50% H2. Experimental gas phase data on Day 0 and 23 are depicted with solid symbols, while modeled values are represented by filled symbols (all the simulated and experimental data illustrated in Figure 5 and Supplementary Figures S6, S7 are reported in Supplementary Table S9). The figures also illustrate experimental trends for pressure and VFAs, which are shown with filled symbols, whereas the simulated trends are reported with dashed lines.

**Figure 6:**
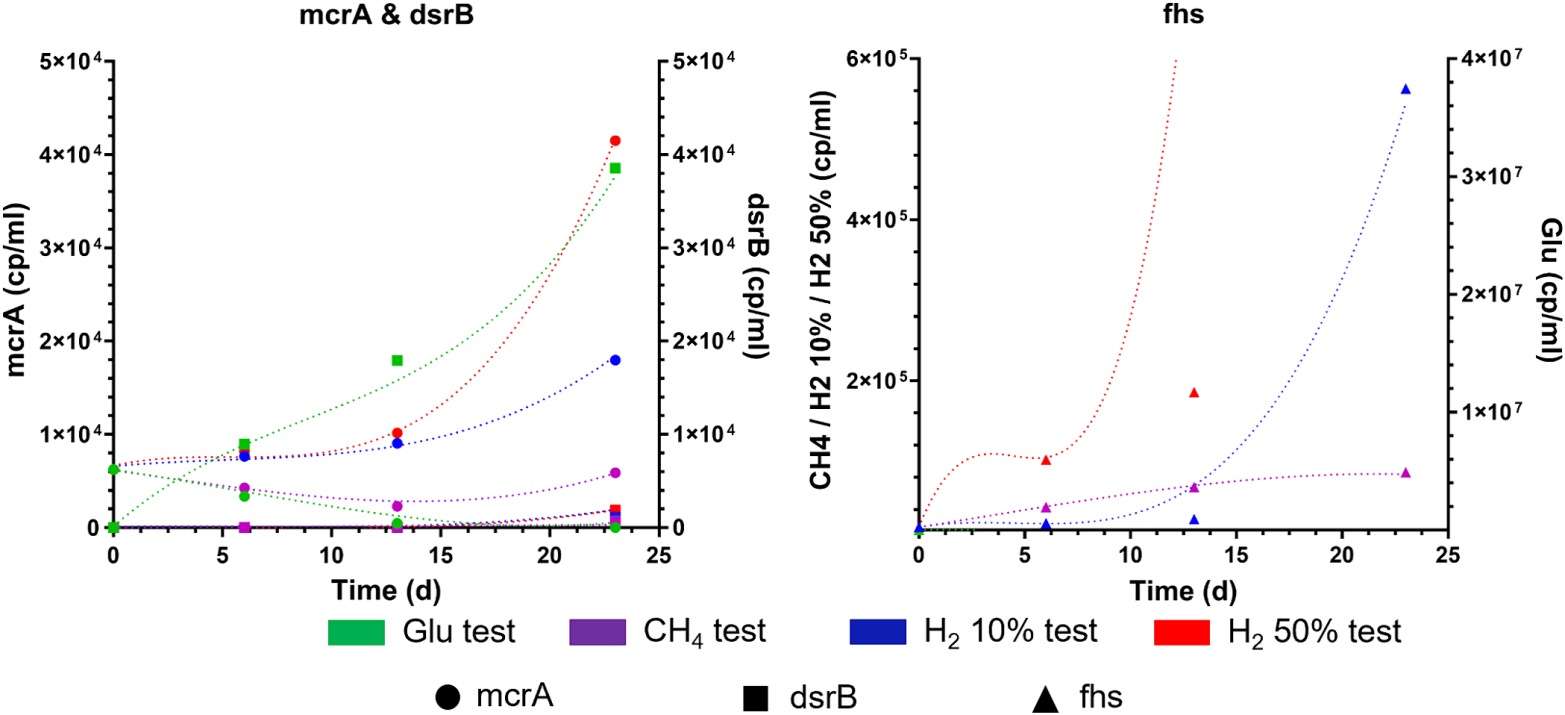
Experimental (filled symbol) and simulated trends (dash lines) of copies/ml of *mcrA*, *dsrB* and *fhs* during batch cultivation of R1 formation waters under different CH_4_/H_2_ gas mixtures.

Results of the simulations indicates that the trends of pressure decreases are similar in almost all the analyzed cases and are mainly due to gas and liquid sampling and the dissolution of gases in the liquid culture, confirming the accuracy of the generated model. The model also provides information on the variation of moles present in the gas phase for each component. In particular, small H_2_ productions are recorded in cases with glucose or 100%CH_4_ only for R1 and R3. Slight consumption of hydrogen was recorded for all cultures, when H_2_ was initially injected in the headspace. For all four reservoirs hydrogen consumption was almost negligible (< 0.1%) for H_2_ 10%. Instead, in cases with H_2_ 50%, the simulations report hydrogen consumptions around 1.2–1.6% compared to the initial moles for R1 and R3, and less than 0.2% for R2 and R4. As for CH_4_, the simulations do not show any production in R2 and R4 in all four case studies. For R1 and R3, a small production of methane is recorded, less than 0.003% relative to the initial moles present, only in cases where H_2_ is present from the beginning, particularly in the case with 50% of H_2_. Such results obtained from the modeling seemingly highlight higher microbial activity in R1 and R3 when compared to R2 and R4, in accordance with the experimental results. Similar trends are observed when analyzing the growth kinetics of HM, AB, and SRB modeled with the growth model (Supplementary Table S1). The results shown in Figure 6 for R1 and Supplementary Figure S8 for R2, R3, R4 in the Supplementary material display the simulated trends of cell number growth for the three considered microbial classes.

**Figure 7:**
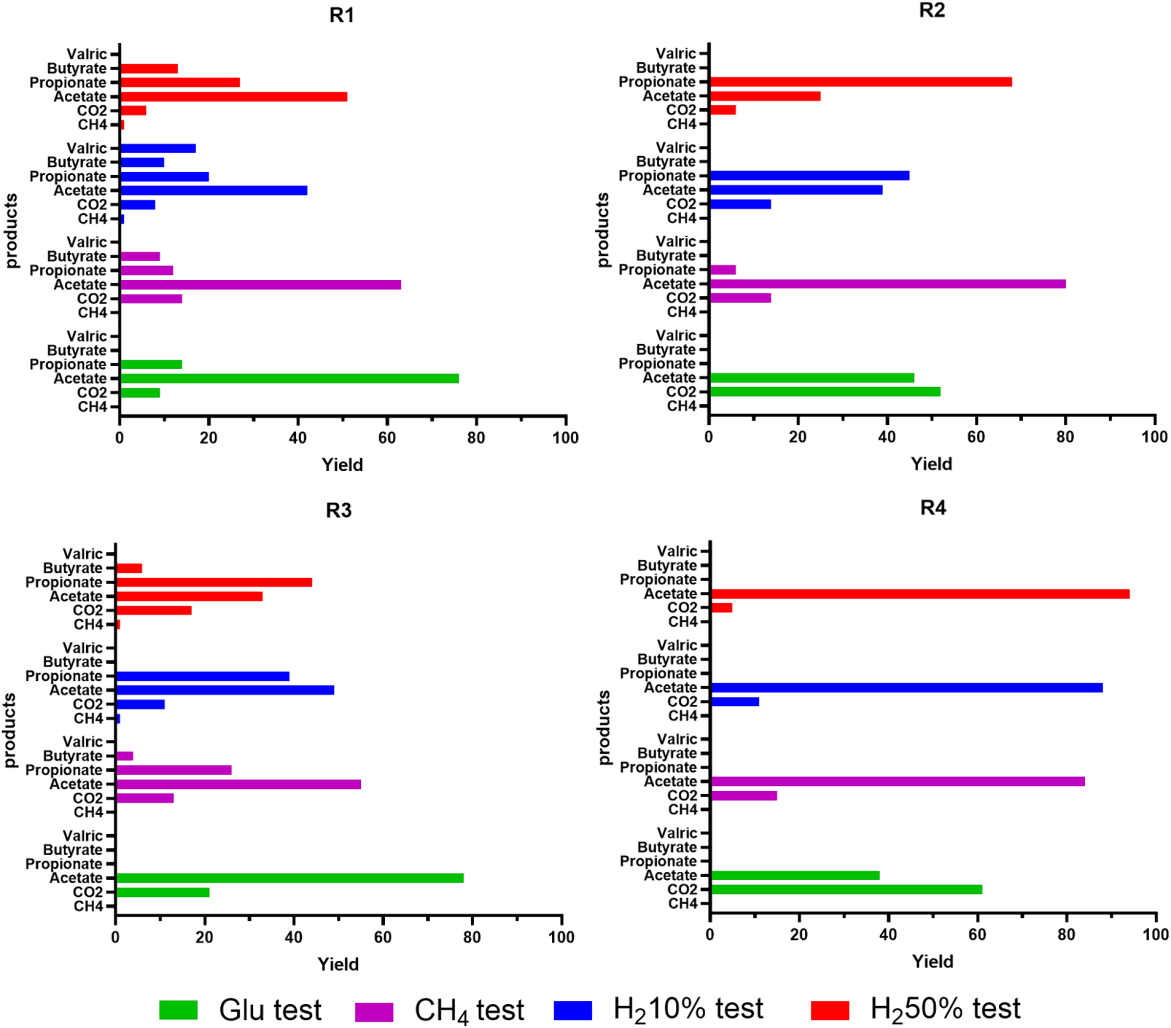
Simulated trends of carbon selectivity (Yield) after 23 days of batch cultivation of R1-R2-R3-R4 formation waters under different CH_4_/H_2_ gas mixtures.

Modeled results align with those obtained experimentally, emphasizing that AB are the most active microbial cluster in formation waters from R1, R2, R3 and R4. Regarding methanogenic metabolisms, a slight growth in cell numbers is recorded only for R1 and R3 in cases with H_2_ 50% and H_2_ 10%, but it is much lower than the values obtained for AB. Low microbial activity and cell number growth was also found for SRB, which report even lower levels than HM. The discrepancy in the growth levels of microbial classes seemingly points out minimal HM and SRB activity in the investigated reservoirs. Throughout the simulations, the monitoring of microbial activity confirmed that R2 and R4 have shown lower or decreased microbial growth compared to R1 and R3. Dominant presence of AB was observed in all four reservoirs, resulting particularly remarkable in R1 and R3. This dominance relates to the increased production of various acidic species, such as acetate. This predominance of acetogenic metabolisms is made more evident when analyzing the quantities of VFA produced, along with their mass balances, carbon and hydrogen uptake, as well as the selectivity of carbon and hydrogen distribution metabolized by microbes in various products generated by the three selected microbial classes, mainly CH_4_, VFA, CO_2_, and H_2_S. Carbon uptake in moles of CH_4_, CO_2_, acetate, propionate and butyrate and hydrogen uptake in VFA, H_2_S, CH_4_, as calculated by the model are presented in Supplementary Tables S10, S11 of the Supplementary material. From the simulation results of considered cases, the majority of carbon is utilized for VFA production, whilst the remaining part is used for CO_2_ production. Only in certain cases a minimal part is utilized for methane production, specifically for R1 and R3 in cases with H_2_ 50% and H_2_ 10%. The model reproduces a similar type of partitioning for hydrogen as depicted in Supplementary Table S3. As with carbon, most of the uptaken hydrogen is utilized for the production of VFA, with only a minimal part (<1%) being used for the production of methane and H_2_S. Particularly for H_2_S, in cases where hydrogen is not provided in the headspace gas mixture, simulations show minimal usage of acetate for its production. Higher uptake of carbon and hydrogen is observed in R1 and R3 in cases were H_2_ was initially present in headspace gas mixture (i.e., H_2_ 10% and H2 50%). A direct consequence of hydrogen and carbon uptake data is the selectivity data reported in Figure 7, for all four reservoirs at the end of fermentation.

For R1 amended with glucose, carbon is divided into 10% in CO_2_, and the remaining 90% in VFA; in the case with 100%CH_4_, about 15% goes to CO_2_ and 85% to VFA. We have similar trends also for cases with 10%H_2_ and 50%H_2_, with carbon percentages destined for VFA greater than 90% and less than 1% for CH_4_, which is present only in these two case studies. Also, the variability of generated products in terms of VFA increases for case studies with hydrogen present from the beginning. The results obtained for R3 are very similar to those obtained for R1 in terms of carbon division between CO_2_, CH_4_, and VFA. Unlike in R1, there is greater selectivity toward CO_2_, always greater than 12%, and lower selectivity toward acetate (about 10–20% less than R1), mainly due to the higher production of other VFAs such as propionate, derived from acetate itself (Supplementary Table S1). For instance, for R3, in the case with H_2_ 50%, there is about 17% in CO_2_, 33.5% in acetate, 44.3% in propionate, 6.6% in butyrate, and less than 1% in CH_4_.

For R2 and R4, the simulation results tell us that in glucose cases, more than half of the carbon uptake ends in CO_2_, the remaining part in acetate. In H_2_ 10% cultures, about 15% in CO_2_ and the remaining part in acetate and propionate, and in cases with H_2_ 50% about 5% in CO_2_ and the remaining part in VFA. Similarly, selectivity of hydrogen uptake, points out that almost all hydrogen is used for VFA production, whilst less than 1% is used for production of H_2_S and CH_4_ in traces. Particularly for H_2_S, a more accentuated presence is noticed when H_2_ is initially present in tested gas mixtures. Extending the simulation time of the models from 23 days corresponding to the experimental time up to 90 days of simulated time provides information on what could happen to the bioprocesses until various limitations such as lack of nutrients or carbon or sulfate sources come into play. The results on the selectivity for carbon and hydrogen at 90 days are reported in Supplementary Figure S12 in the Supplementary material. As can be seen from the simulation results, the selectivity concerning hydrogen remains almost unchanged. The selectivity concerning carbon instead shows slightly different values compared to those obtained on 23 days. In particular, the selectivity concerning VFA generally increases (about 10%), and among the VFAs themselves, the selectivity toward acetate decreases in favor of other fatty acids such as propionate. For what concerns the trials that do not consider the addition of nutrients to the liquid phase, the simulation data are in line with the experimental results, underlining absence of activity and growth for the considered microbial clusters (i.e., AB; HM; SRB).

## 4 Discussion

### 4.1 Batch enrichment of reservoir formation waters

#### 4.1.1 Characteristics of the inoculant

In a previous work Bassani et al. (2023) characterized the formation waters from four Italian NG reservoirs at hydro-chemical and metagenomic level, obtaining information regarding the presence of metabolic pathways within the indigenous microbial communities that could be of interest when considering UHS strategies through predictive analysis of functional gene categories. Although our previous study highlighted the presence of low organic carbon sources (R1 and R3), limited concentrations of nutrients involved in cells proliferation and basic functions (i.e., PO_4_^−^ < 8 mg/L) and high salinity >40 g/L (R2 and R4), potentially limiting microbial growth (Gerardi, 2003; Romero-Güiza et al., 2016), the literature reports that different orders of microbes involved in acidification, souring and bio-corrosion phenomena, such as AB and SRB, might still be able to proliferate in underground formations (Muyzer and Stams, 2008; Gniese et al., 2014; Panfilov et al., 2016; Dopffel et al., 2021). Moreover, it has been largely described that hydrogenotrophic organisms like HM, SRB and homoacetogenic bacteria could affect H_2_ storage causing loss of the injected H_2_ (Panfilov, 2010; Gniese et al., 2014). For such reasons, in this work, we investigated the microbial activity and growth of ABs, SRBs and HM present in formation waters of reservoirs R1, R2, R3 and R4 (Bassani et al., 2023) when cultivated without and with full provision of nutrients and co-factors, under different headspace atmospheres emulating mixtures of interest for UHS.

In contrast to qualitative metagenomics analysis, quantitative qPCR on targeted gene sequences allows one to enumerate different microbes in the samples. In this study, the characterization of the inoculant using qPCR clearly gives an indication of the relative density (copies/ml) of HM, SRBs and AB populations of the four formation waters. Our results highlighted that AB, targeted by *fhs* gene, are the predominant microbial cluster in formation waters samples from R1, R2 and R3. These findings are in agreement with the occurrence of microorganisms belonging to the *Synergistales*, *Thermotogales* and *Clostridiales* orders, whose metabolisms are related to both acido- and acetogenesis, previously identified in the formation waters assessed (Bassani et al., 2023). Similarly, different orders of HM were identified by both bacterial 16S and *mcrA* sequencing in all reservoir formation waters, nonetheless while enumeration of HM is possible in both R1 and R3, the methanogenic populations of R2 and R4 appear to be considerably under-represented and below detection levels. Indeed, the enumeration of the SRB *dsrB* gene in the initial inoculant reveals low numbers of *dsrB* copies/ml in all reservoirs with values frequently being below the threshold of detection in all inoculants (Table 3).

qPCR analysis performed on the inoculation material clearly displays populations characterized by a low microbial abundance of both methanogens and SRB compared with those found in other studies performed on different geological formations, where methanogenic or sulfate reducing activity has been detected (Vigneron et al., 2017; Ranchou-Peyruse et al., 2019; Buriánková et al., 2022). Indeed, for HM, Buriánková et al. (2022) reported that natural densities of *mcrA* sequences between 1.84 × 10^4^–2.4 × 10^7^ copies/ml in different reservoirs where active methanogenesis was detected. Ranchou-Peyruse et al. (2019) found concentrations of *dsrB* copies/ml between 10^2^ and 10^4^ in subsurface aquifers where H_2_S production was observed, whilst Bomberg et al. (2015) found similar densities in deep bedrock fracture fluids. This difference from our results on the quantification of *mcrA* and *dsrB* gene copy numbers in the above-mentioned studies suggests that, in the reservoirs under investigation, these two microbial classes of fundamental interest from the perspective of the UHS are not/are only minimally active.

#### 4.1.2 Microbial growth and activity during batch cultivation test

Monitoring of microbial population of the four reservoirs during the whole experiment (day 23) confirms the dominant presence of AB in R1, 2, 3 and 4. This is particularly evident in both R1 and R3 with the increase in *fhs* copies/ml occurring simultaneously with the formation of different acid species in all tested conditions with acetate being the most represented scVFAs (Figures 3, 4). Acidogenesis and acetogenesis are common phenomena occurring in both anthropic and natural anaerobic environments and are involved in the degradation of organic matter (Gerardi, 2003; Miranda-tello et al., 2004; Hattori, 2008; Molíková et al., 2022). In particular, acetogens are a widespread group of microorganisms capable of metabolizing different organic compounds (i.e., scVFAs, organic acids, carbohydrates) leading to the production of acetate as the main metabolic product. Furthermore, homo-acetogenic organisms can ferment acetate from CO_2_ and H_2_ (Drake and Daniel, 2004; Drake and Küsel, 2005; Drake et al., 2006; Ragsdale and Pierce, 2008; Huang et al., 2012). Although the rate of acetogenesis and homo-acetogenesis was not determined, the provision of fermentable carbohydrates (i.e., glucose, yeast extract, peptone) and thermodynamic of the reaction (Table 1) could have favored fermentative metabolisms over homo-acetogenic ones. Such a hypothesis is also sustained by the increase of CO_2_ in headspace gases recorded on day 23.

Although also in R2 and R4 predominant acetogenic organisms and metabolisms were previously identified (Bassani et al., 2023), in the present study the quantitative analysis based on qPCR reveals lower or reduced growth of the microbial cluster when compared to R1 and R3 cultures. A possible reason for the lower acetogenic density in R2 and R4 cultures could be related to the high salinity levels previously reported for the two formation fluids (>40 g/L). Indeed, different studies report that high salt concentrations, typical of underground geological formations, act selectively on indigenous microbial populations decreasing their diversity and specifically reducing acetogenic degradation of organic compounds and increasing rate of homo-acetogenesis (Gniese et al., 2014; Berta et al., 2018; Dopffel et al., 2021), which could explain the reduced signs of acetogenic growth in R2 and R4 cultures.

HM belonging to the *Methanobacteriales* and *Methanomicrobiales* orders mostly rely on CO_2_ reduction to CH_4_ by using H_2_ as the main electron donor (Table 1) were previously identified in reservoir formation waters used as inoculant in the present study (Bassani et al., 2023). In the natural environment, substrates for hydrogenotrophic methanogenesis, namely CO_2_ and H_2_, are generated during various steps of organic matter degradation. As an example, different studies frequently report interactions between HM and homo-acetogenic bacteria capable of using the Wood-Ljungdahl pathway (WLP) in the opposite direction by oxidizing acetate to CO_2_ and H_2_ which can be reduced to CH_4_ by HM (Hattori, 2008; Manzoor et al., 2018; Timmers et al., 2018). Moreover, in underground geological formations, carbonates dissolved in formation waters could provide the necessary CO_2_ for HM with the supply of H_2_ being provided from other microbial processes (Nazaries et al., 2013; Dopffel et al., 2021; Bellini et al., 2022); The literature also defines clearly the effect that H_2_ external provision has on HM with several studies reporting an increase in their abundances (Bassani et al., 2015; Rachbauer et al., 2016, 2017; Treu et al., 2018).

In the present study, no relevant increases occur in culture methanogenic populations of both R1 and R3 positive control samples and enrichment cultures with 100%CH_4_ in headspace gas, despite the availability of carbonates present in both formation waters and the provided nutrient media, with acidogenic and acetogenic activity leading to CO_2_ release in the headspace (Figures 3, 4). On the other hand, both R1 and R3 cultures growing with 10 and 50% H_2_ in headspace gas display higher and increasing HM density during the experiment (Figure 4). Along with the information provided by the literature, these results point out the fact that although different sources of CO_2_ are available, the absence of H_2_ in sufficient amounts clearly limits the growth of HM. Despite the evident signs of HM growth in R1 and R3 cultures under 50% H_2_ headspace, quantification of their main metabolic product was not possible due to the covering effect exerted by the CH_4_ already present in the gas mixture. Although no relevant changes in either H_2_ or CH_4_ headspace concentrations are revealed by GC analysis, both R1 and R3 cultures grown under 50% H_2_ headspace displays the highest ΔP (R1: 0.13 bar, R3: 0.1 bar) which could be potentially attributed to HM competing with homo-acetogenic bacteria for H_2_ (Figure 4).

In contrast to R1 and R3 cultures, there is a reduced or complete absence of HM growth in those seeded with R2 and R4 formation waters, respectively. With HM generally relying on interactions with other microorganisms for their supplies of CO_2_ and H_2_, and with a reduction of reaction rates generating them being promoted by high salinity, HM growth is likely limited. Furthermore, as previously discussed the possible presence of homo-acetogenic organisms could cause competition for H_2_, with the low-population density of HMs favoring the proliferation of more represented and abundant competitors.

Along with AB and HM, several studies report the presence of SRBs in different geological formations such as oil fields, sulfate and CH4-rich groundwater from deep boreholes, marine sediments, aquifers and salt mines (Martini et al., 2005; Mochimaru et al., 2007; Morozova et al., 2011; Bomberg et al., 2015; Varjani and Gnansounou, 2017; Rusmanis et al., 2019; Rizzo et al., 2020; Briones-Gallardo et al., 2022). Although initially believed to rely only on H_2_ for sulfur reduction, different studies characterized SRBs as capable of generating electrons from the oxidation of organic compounds including sugars, organic acids (i.e., scVFAs, acetate) and amino acids. Furthermore, in natural environments, SRBs usually compete with HM and AB for H_2_, with the presence of available sulfate being crucial for SRB (Muyzer and Stams, 2008; Plugge et al., 2011). We previously reported in the same formation waters the presence of different microorganisms related to the biological reduction of sulfur compounds (i.e., orders *Thermotogales*, *Synergistales, Haloanaerobiales and Deferribacterales*) (Bassani et al., 2023). However, the measurement of microbial densities in the reservoir formation waters used for batch cultures clearly showed that SRBs are the least represented group of microorganisms compared to AB and HM, with the lower density possibly related to the low sulfate content (<50 mg/L) measured in all reservoirs fluids investigated (Bassani et al., 2023). During the batch cultivation assays, although sulfate and different C sources have been either provided in the enrichment media (i.e., acetate, yeast extract, peptone and glucose) or generated by the metabolic activities of other microorganisms (i.e., scVFA), the monitoring of the density of SRB population mostly confirms the occurrence of low values, related to reduced or non-significant growth for most of the tested conditions. Significant signs of growth were reported in R1 cultures, R2 and R3 samples amended with H_2_ 50%. Nonetheless, despite the detected growth of SRB, presence of H_2_S was only detected in R1 positive control samples with gas concentrations reaching a maximum of <0.05% by day 23 (Figure 4). Given the displayed results it is possible that in tested conditions both inter-species competition for substrates and the use of produced H_2_S by other microbial clusters limited production of the corrosive gas species (Miranda-tello et al., 2007; Nyyssönen et al., 2012; Allen et al., 2015; Bomberg et al., 2015; Oliveira et al., 2022).

In the present study the initial low densities of SRB populations, together with the lack of sulfate substrates in the formation waters analyzed, is probably the main cause of the reduced sulfate reduction activity found in the present study, which appears considerably lower than other example reported by the literature (Ranchou-Peyruse et al., 2019; Haddad et al., 2022). Although the experimental conditions aimed to stimulate microbial growth through nutrient supply, with initial SRB concentrations on average lower than those of both HM and AB, the latter could be favored by a higher density in the competition for substrates, with SRBs relying on the supply of metabolic products of predominant AB, as in R1 samples.

### 4.2 Effects of microbial activity on gas mixtures of interest for UHS

Given the importance that H_2_ has in the program for full decarbonization by 2050, the development of valuable storage strategies is a paramount objective. In this context, UHS strategies gained interest due to the availability of large storage volumes in underground formations (Reitenbach et al., 2015; Verga, 2018; Pan et al., 2021; Muhammed et al., 2022). With several studies underlining the presence of specific microbial communities that might affect underground gas storage, a better understanding of their activity is required (Gniese et al., 2014; Valk et al., 2020; Molíková et al., 2022).

In the present study, we showed the presence of microbial activity in formation waters collected from four depleted gas reservoirs of potential interest from the perspective of UHS. However, the testing conditions were different from the actual reservoir conditions (e.g., pressure and nutrient supply), yet the activity was very limited.

#### 4.2.1 Acidogenesis and acetogenesis

In particular, acetogenic and acidogenic microorganisms appear to be most relevant among the targeted microbial groups (i.e., AB, HM and SRB). Both acetogenesis and acidogenesis occur from the degradation of organic substrates leading to the production of different scVFAs, CO_2_ and H_2_ (Gerardi, 2003; Drake and Daniel, 2004; Drake and Küsel, 2005; Drake et al., 2006; Ragsdale and Pierce, 2008; Molíková et al., 2022). With the supply of nutrients (i.e., glucose, yeast extract, peptone) stimulating fermentative metabolisms, a release of CO_2_ has been observed in all cultures in which growth and activity of AB have been detected (i.e., R1, R2, R3), resulting in minimal dilution of the gas mixtures in the headspace (Figures 3, 4).

#### 4.2.2 Methanogenesis

HM are considered of interest for UGS/UHS due to their use of H_2_ for the reduction of CO_2_ to CH_4_ (Liu and Whitman, 2008). During enriched batch culture assays, the growth of HM was detected in only two of the four formation waters used in the present study. Results obtained from R1 and R3 cultures clearly display the dependence of HM growth on the availability of H_2_ and the increase in its concentrations (Figure 4). Although it was not possible to measure produced CH_4_ due to the masking effect with the headspace gases concentration remaining unvaried, the minimal losses in headspace pressure observed in cultures cultivated under 50% H_2_ can address a partial consumption of H_2_ related to HM activity. It is worth mentioning some aspects related to the activation of HM in the present work. First, in contrast to other studies (Buriánková et al., 2022; Haddad et al., 2022), during enrichment nutrients that are not naturally present in formations waters (i.e., phosphate, vitamins) are supplied. Second, the initial HM population density in formation waters before enrichment appears to be two to five times lower than in other studies reporting methanogenic activity in different underground formations; (Ranchou-Peyruse et al., 2019; Buriánková et al., 2022; Haddad et al., 2022). Whilst the presence of additional and specific nutrients in batch cultures surely stimulates HM growth and activity, their absence in the real environment could result in limiting conditions. Furthermore, only after enriched cultivation assays, the HM populations reach densities similar to those previously reported in the literature for active methanogenic populations in underground formations (Ranchou-Peyruse et al., 2019; Buriánková et al., 2022). This considered, it could be hypothesized that in real-life conditions the activation of the HM community could be limited when considering the reservoirs under investigation.

Finally, it is worth mentioning that, although the presence of methanogenic communities in underground formation could lead to losses of stored H_2_ within the logic of UHS, from a different perspective, the biological conversion of H_2_ into CH_4_ could be seen in a positive light when considering the possibility of underground methanation (Bellini et al., 2022). Methanation in deep geological structures is a well-reported microbial process which if properly enhanced through the injection of H_2_:CO_2_ blends would allow the use of such geological structures as underground methanation reactors (UMR) characterized by a considerable working volume. In this concept, either indigenous or introduced HM populations would act as bio-catalyst operating the reduction of CO_2_ to CH_4_ by consuming H_2_ (Panfilov et al., 2016; Amez et al., 2021; Bellini et al., 2022; Molíková et al., 2022). Although deep saline aquifers, salt caverns and depleted hydrocarbons reservoirs have been considered for UMR, only three projects have demonstrated the possibility of developing UMR technologies in NG reservoirs (Flemisch et al., 2011; Pérez et al., 2016; RAG, 2018; Strobel et al., 2020; Nikolaev et al., 2021; Molíková et al., 2022).

#### 4.2.3 Sulfate reduction

As for HM, SRB are of interest in both UGS and UHS strategies due to their consumption of H_2_ and relative production of corrosive H_2_S, which could lead to biological corrosion phenomena. In a natural environment, SRB usually competes with HM for H_2_ with sulfate reduction being favored in the presence of SO_4_^3−^ due to the thermodynamics of the reaction (Toleukhanov et al., 2015; Michas et al., 2020; Valk et al., 2020; Haddad et al., 2022). Nonetheless, in the reduced presence of SO_4_^3−^ and due to their metabolic flexibility SRB have been demonstrated capable of growing through fermentative processes (Miranda-tello et al., 2004; Muyzer and Stams, 2008; Bomberg et al., 2015). Although Bassani et al. (2023) previously identified SRB in formation waters used in the present study, the initial density of the microbial population appears to be considerably lower than other example previously reported in similar studies (Nyyssönen et al., 2012; Bomberg et al., 2015; Ranchou-Peyruse et al., 2019), with copies/ml of *dsrB* target gene frequently being below their limit of detection (<1.85 × 10^2^ copies/ml). Similarly to SRB densities, sulfate concentrations reported in the four reservoirs are considerably lower than those reported in the literature for other geological structures considered as possible storage site. Thaysen et al. (2021) mapped several candidate UHS sites considering the sulfate content as a key indicator for the microbial risk assessment associated to H_2_ storage. Considered sites classified by low or high risk displayed SO_4_^3−^ levels between 110 and 3,037 mg/L. With SO_4_^3−^ being essential for the SRB metabolism, low concentrations measured (<50 mg/L) in the four reservoir object of this study seemingly points out at limiting conditions that could reduce the risk associated to SRB activity.

Furthermore, whilst the growth of SRB was detected in different cases during enrichment culture assays, production of H_2_S was detected in traces only in a few cases (e.g., R1 positive control supplemented with glucose; H_2_S: <0.05%), despite the substrates for SRB metabolism having been provided either in the enrichment media (e.g., SO_4_^3−^, carbohydrates) or in headspace gases (e.g., H_2_). Along with the low SRB population densities reported in the inoculants, the absence or reduced growth points out a possible competitive disadvantage of the SRB population when compared to both AB and HM. Both the literature and results obtained in the present study seem to underline the use of fermentative metabolisms by SRB populations, nonetheless, low densities and possible competition for fermentable substrates with more represented AB could have limited SRBs growth and activity in the present study. Adaptation to a more fermentative metabolic attitude and reduced reliance on SO_4_^3−^ reduction by cultured SRB could also have reduced the competition with HM allowing for a more pronounced growth of the latter as seen in R1 and R3. A combination of such factors leads to limited or inexistent modifications to the composition and volume of gas mixtures tested in the present study.

### 4.3 Biogeochemical modeling

Modeling of microbial risks associated with UHS encompasses a multitude of physical, chemical, and biological processes. In particular, hydrogenotrophic microbial processes play a significant role in UHS (Hagemann et al., 2016; Jin and Kirk, 2016) with the integration of flow and transport of nutrients, microbial population dynamics, and biochemical reactions being essential for the model’s final derivation.

Analyzing the production trends of acetogenic, methanogenic, and sulfate-reducing metabolisms offers crucial insights into microbial interactions and their overall impact on the efficacy and sustainability of hydrogen storage processes. A thorough model-based evaluation of these metabolic pathways allows for a nuanced understanding of microbial contributions, potential challenges, and enhance the performance of UHS strategies. In the present study a modeling approach was applied to microbial cultures seeded with formations water from reservoir R1, R2, R3 and R4 with the modeled results being scrutinized and compared with the experimental data reported in this work. Overall, data derived from the modelling are in accordance with the experimental ones, highlighting for the considered microbial populations a predominant activation of AB when compared to HM and SRB when external nutrients are provided. With particular regard to H_2_, modeled data defined that highest H_2_ consumption was reported in H_2_ 50% amended cultures with only 1.2–1.6% of the initial H_2_ molar content consumed in R1 and R3, whilst <0.2% was consumed in R2 and R4. Along with its consumption, the model allowed to define H_2_ yields in the different metabolic products (i.e., VFAs, CH_4_ and H_2_S) of the considered microbial clusters. According to modeling data, most of the consumed H_2_ would be redirected to VFA generation with <1% being consumed in methanogenesis and sulfate reduction.

Both experimental and modeling data reported in the present study seemingly underline low microbial effects on stored H_2_ mixtures with the contribution of hydrogenotrophic clusters like HM and SRB being almost negligible in all tested reservoir fluids. According to the data reported in this work, low microbial densities and competition for H_2_ with more active AB could be addressed as the main reason behind low activity displayed by the two hydrogenotrophic clusters.

This hypothesis is further sustained by the data regarding carbon yields for different products of considered microbial metabolisms. In fact, modeled data evidence how AB related metabolisms present the highest carbon yields when compared to HM and SRB, with most of the available carbon being incorporated in AB metabolic products (i.e., VFAs, CO_2_). When conditions without external nutrient provision were simulated by the developed model, results aligned with the experimental data indicating absence of activation of the microbial clusters of interest in UHS. Along with the experimental ones, data obtained from the model seemingly indicate that microbial activation is strongly affected by nutrient availability, with hydro-chemical conditions similar to those found in the reservoir likely limiting the activity of the indigenous microbial communities. Model simulation estimates on the four reservoirs are generally in accordance with the experimental data presented in this work, underscoring how the developed model is grounded in robust foundations and could potentially be extended to field application for a better understanding of the microbial risk associated with UHS.

## 5 Conclusion

**Figure 8:**
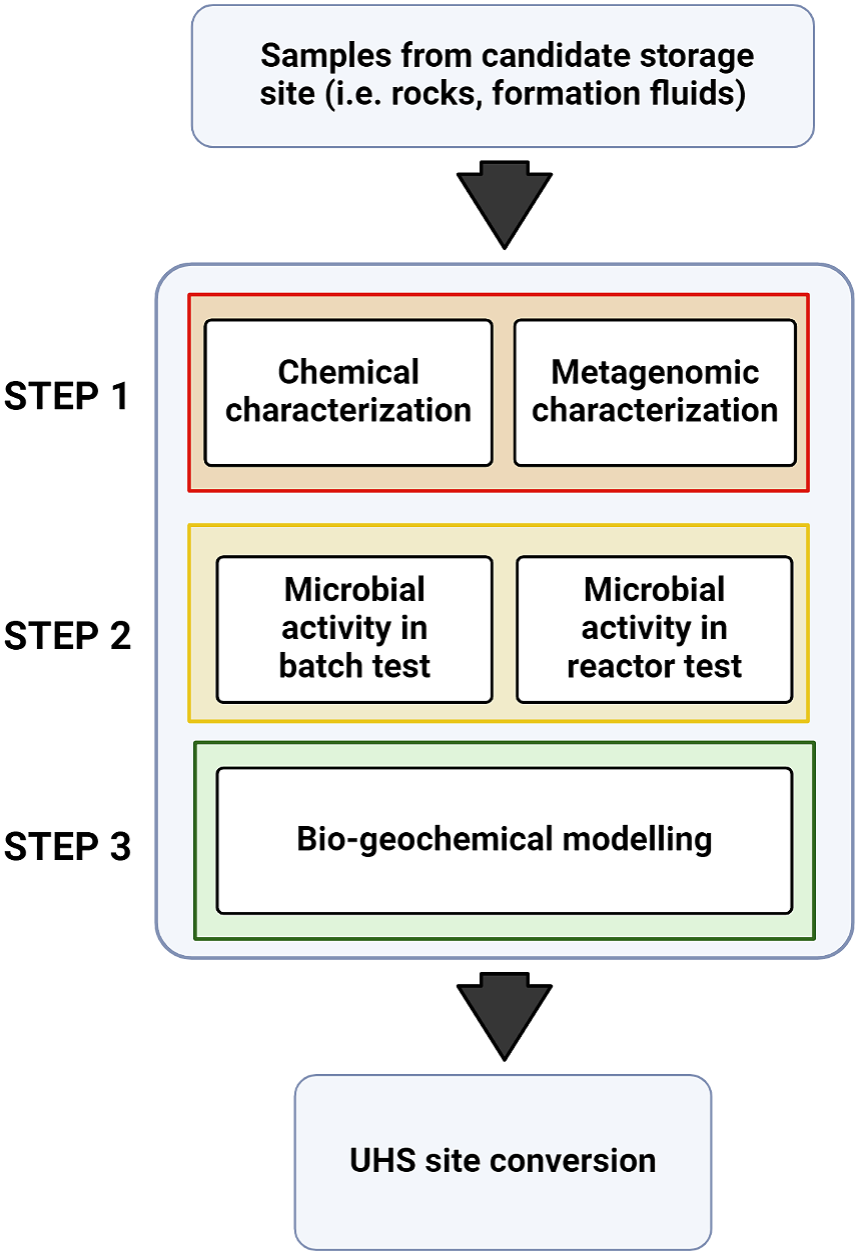
Scheme describing the workflow envised for the assessment for microbial risk associated with UHS.

The procedure described in this study is part of a broader strategy of characterizing and modeling microbial activity in UGSs, which is a key tool to assess the feasibility the feasibility of site conversion to UHS (Figure 8). The first step of this procedure, described in Bassani et al. (2023), aimed at characterizing formation water samples by hydro-chemical and metagenomic analyses. As a second step, the present study aimed to evaluate the growth and effects of different microbial groups, namely HM, SRB and AB, previously identified within the microbial populations of four Italian NG reservoirs, on H_2−_containing gas mixtures. The third step, to be discussed in future works, will be to model the microbial activity on extended timeframes representative of UHSs operations.

The results obtained during the batch cultivation of formation waters of the four reservoirs considered in the present study, namely R1, R2, R3 and R4, showed a dominance of AB populations with fermentation of organic compounds to acetate leading to CO_2_ production causing limited dilution effects on tested gas mixtures. The increasing of H_2_% concentrations in the headspace gases appear, as expected, to stimulate HM growth in R1 and R3. Nevertheless, no relevant changes in the gas mixture concentrations containing H_2_ have been detected, and the minimal headspace pressure reduction that has been recorded could only be partially attributed to the activity of HM as reported by modeling results. Unexpectedly, in the formation fluids of the reservoirs under investigation SRBs are the most under-represented microbial group in both the initial inoculant and the enriched cultures with their effect on tested gas mixtures being not relevant or null. Differences among the activity of the indigenous microbial population of the four reservoirs are mainly attributed to the hydro-chemical characteristics of the formation waters, with high salt concentrations characterizing R2 and R4 (Bassani et al., 2023) likely limiting the growth of the microbial populations when compared to R1 and R3.

Furthermore, obtained results underline how reservoir hydro-chemical conditions could be limiting, with microbial growth and activity being stimulated only upon provision of external nutrients not naturally available in the reservoir. To the best of our knowledge, the present work represents the first attempt at characterizing the microbial activity of preserved samples from depleted gas reservoirs present on the Italian territory, and the experimental data combined with the basic biogeochemical activity model allows one to predict the reservoir-scale behavior of the microbial populations of the reservoir under investigation within the framework of the implementation of UHS.

In future work, tests of microbial activity under pressure and temperature conditions similar to those of the reservoir will be conducted in a customized reactor system to obtain experimental data representative of the underground systems (Vasile et al., 2024). This will enable a better outlook on the effects that indigenous microbial communities may have on the storage of gases in Italian underground geological formations and serve as a reference model at national and international level for the implementation and operation of UHS facilities.

## Data availability statement

The original contributions presented in the study are included in the article/Supplementary material, further inquiries can be directed to the corresponding authors.

## Author contributions

RB: Data curation, Formal analysis, Investigation, Methodology, Visualization, Writing – original draft, Writing – review & editing. NV: Data curation, Formal analysis, Methodology, Software, Validation, Visualization, Writing – original draft. IB: Methodology, Writing – review & editing. AV: Data curation, Investigation, Writing – review & editing. CC: Resources, Writing – review & editing. DB: Resources, Writing – review & editing. MS: Resources, Writing – review & editing. CP: Funding acquisition, Project administration, Supervision, Writing – review & editing. FV: Funding acquisition, Project administration, Supervision, Writing – review & editing. BM: Methodology, Project administration, Supervision, Writing – review & editing.

## Footnotes

^1^ https://unmig.mite.gov.it/stoccaggio-del-gas-naturale/
