# Supplementary material for: Investigating the activity of indigenous microbial communities from Italian depleted gas reservoirs and their possible impact on underground hydrogen storage
Source: Front Microbiol. 2024 Apr 24;15:1392410. doi: 10.3389/fmicb.2024.1392410 (PMC11079786; doi:10.3389/fmicb.2024.1392410)
Supplement: Supplementary file 1 [file Table_1.DOCX]

Supplementary Material

# Supplementary Figures and Tables


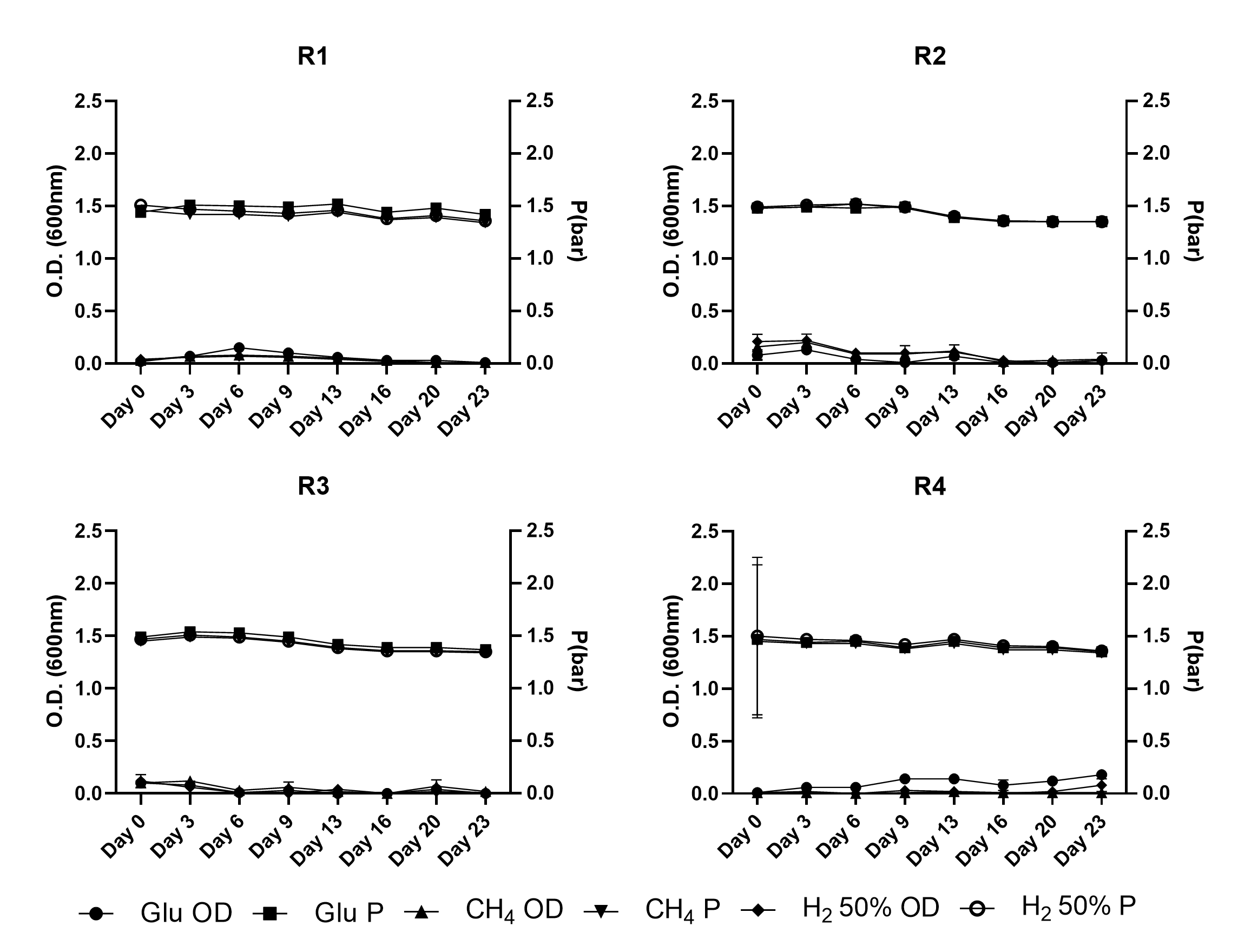


**Supplementary Figure 1 – S1.** O.D. and P variations observed during batch cultivations in absence of additional nutrients for formations water of the four reservoirs object of the present study.


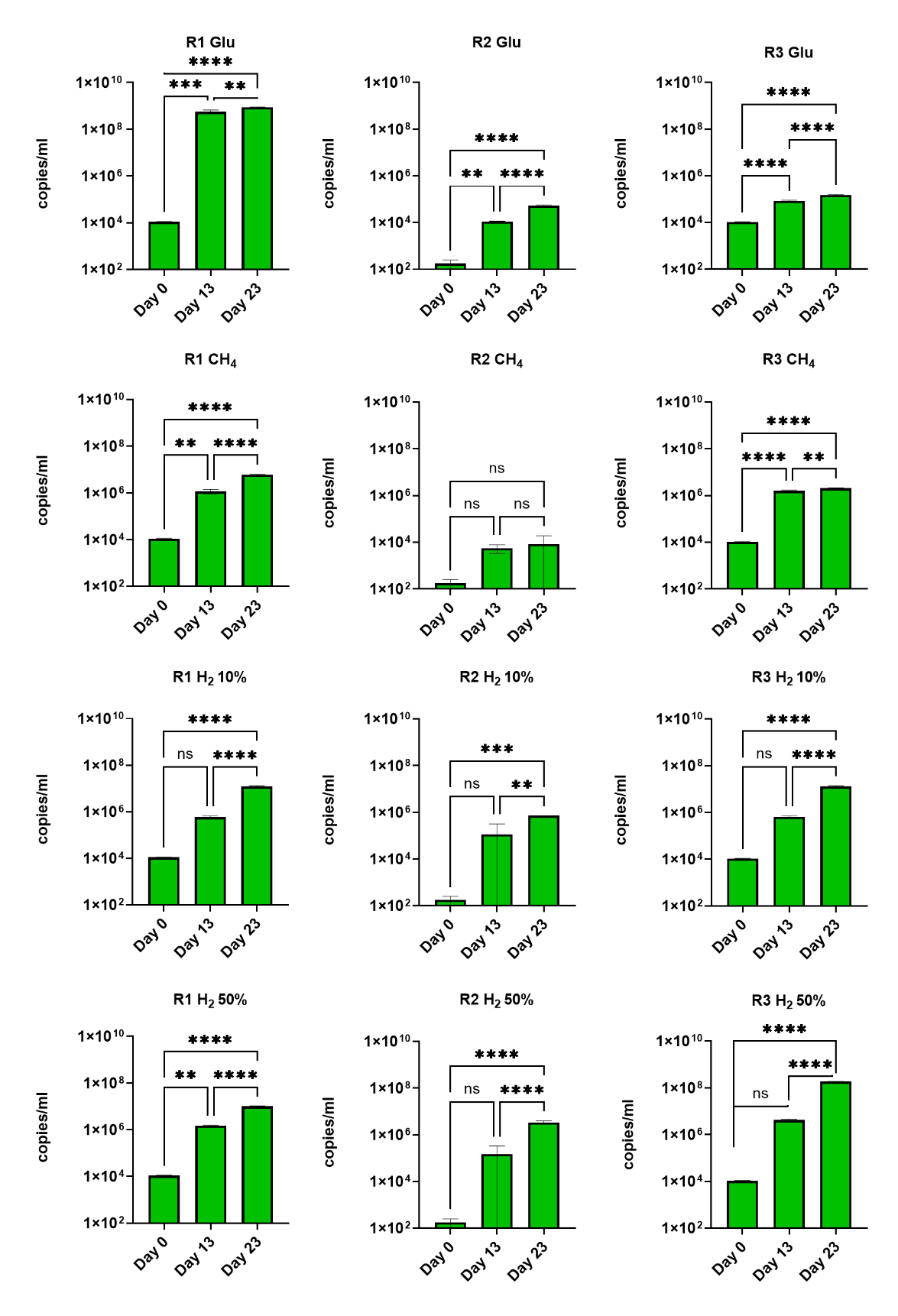


**Supplementary Figure 2 – S2.** One-way ANOVA tests for fhs copies/ml variation in reservoir object of the present study. The results reported in the present work are those with P<0.03.


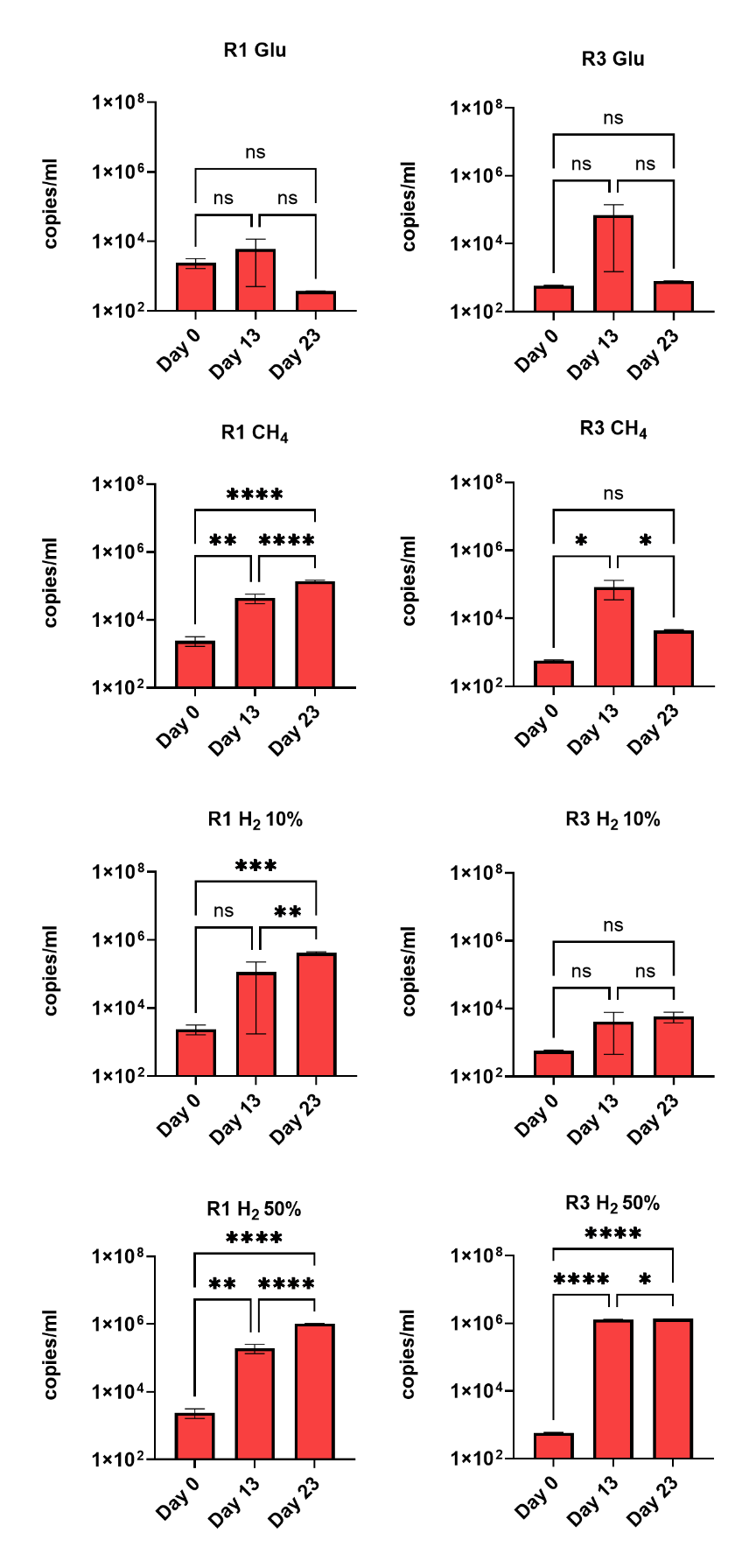


**Supplementary Figure 3 – S3.** One-way ANOVA tests for mcrA copies/ml variations in reservoir object of the present study. The results reported in the present work are those with P<0.03.


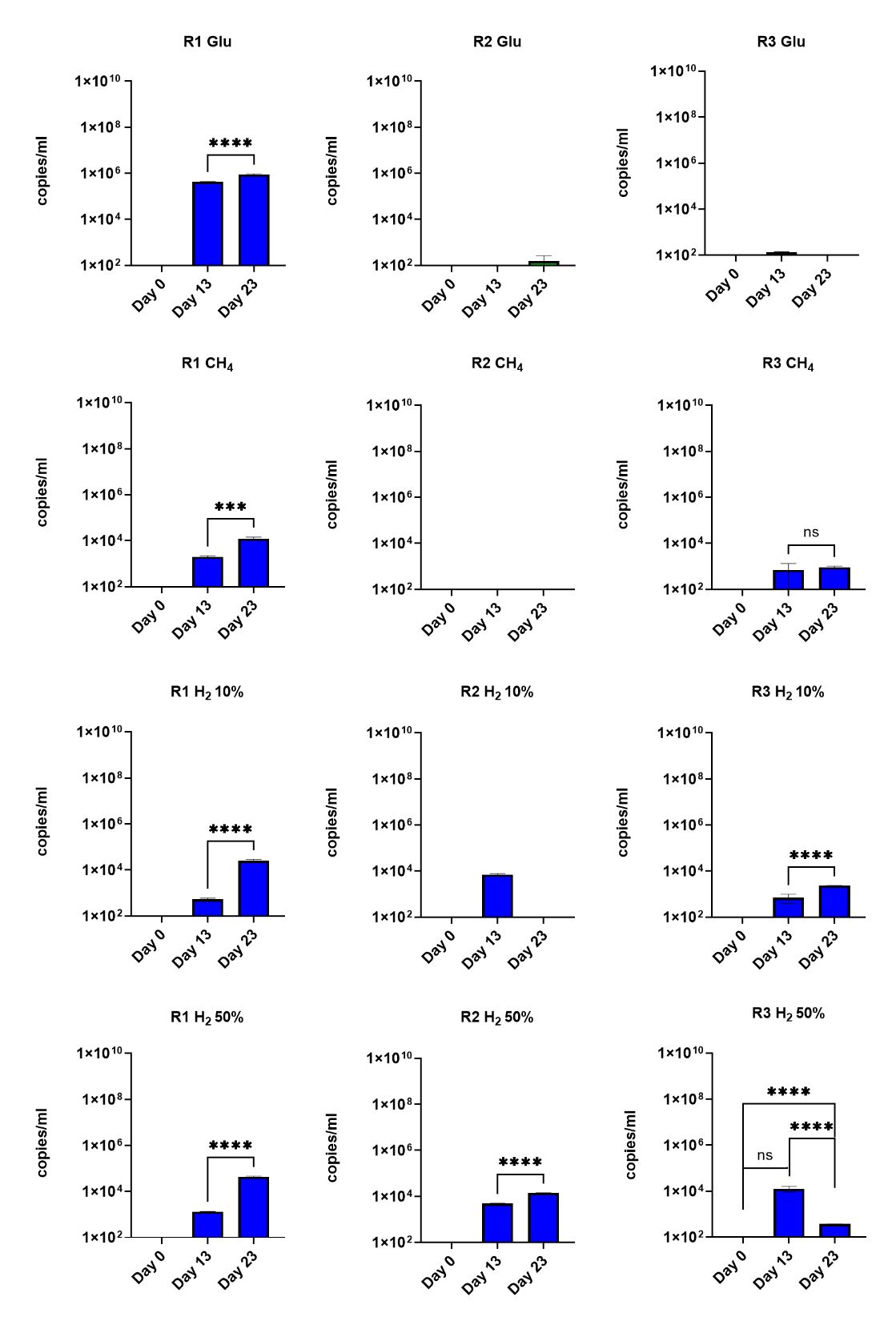


**Supplementary Figure 4 – S4.** One-way ANOVA tests for dsrB copies/ml variations in reservoir object of the present study. The results reported in the present work are those with P<0.03.

Supplementary Table 1 – S5: Main equations implemented in the model (follows in next page)

|  | ***Equations*** |
| --- | --- |
| **Sulphate-reducing reactions** | 4 H_2_ + SO_4_^2–^ + H+ → HS^–^ + 4 H_2_O |
|  | Acetate^–^ + SO_4_^2–^ → 2 HCO^–^ + HS^–^ |
|  | Propionate^–^ + 0.75 SO_4_^2–^ → Acetate^–^ + HCO_3_^–^ + 0.75 HS^–^ + 0.25 H^+^ |
|  | Butyrate^–^ + 0.5 SO_4_^2–^ → 2 Acetate^–^ + 0.5 HS^–^ + 0.5 H^+^ |
| **Acetogenic reactions** | 2CO_2_ + 4H_2_ → CH_3_COOH + 2H_2_O |
|  | Glucose → 3 Acetate^–^ + 3H^+^ |
|  | Propionate^–^ + 3 H_2_O → Acetate^–^ + HCO_3_^–^ + H^+^ + 3 H_2_ |
|  | Butyrate^–^ + 2 H_2_O → 2 Acetate^–^ + H^+^ + 2 H_2_ |
| **Methanogenic & Homoacetogenic reactions** | CO_2_ + 4H_2_ → CH_4_ + 2H_2_O |
|  | Acetate^–^ + H_2_O → CH_4_ + HCO_3_^-^ |
|  | 4 H_2_ + HCO_3_^–^ + H^+^ → CH_4_ + 3 H_2_O |
|  | 4 H_2_ + 2 HCO_3_^–^ + H^+^ → Acetate^–^ + 4 H_2_O |
| **CO_2/_Bicarbonate equilibrium** | CO_2,g_ + H_2_O ⇄ CO_2,liq_ + H_2_O ⇄ H^+^ + HCO^3-^ |
| **H^+^/H_2_ equilibrium** | 8H^+^ + 8e^−^ → 4H_2,liq_ |
| **HS^-^/H_2_S equilibrium** | H_2_S ⇄HS^-^ + H^+^ |
|  | HS^-^ ⇄ ${S_{2}}^{-}$ + H^+^ |
|  | ${S_{2}}^{-}$ + 2H^+^ ⇄ H_2_S |
|  | |
| **Species mass balance** | $\frac{d\left( c_{j}V_{r} \right)}{dt}=V_{r}R_{i}$ |
|  | $R_{i}= \sum_{j} v_{ij}r_{j}$ |
|  | |
| **Ideal gas law** | $p=R_{g}T\sum c_{j}$ |
|  | |
| **Mass action law** | $r_{j}=k_{j}^{f}\prod_{i \in react} c_{j}^{{-v}_{ij}}$ |
| **Arrenius expression** | $k^{f}=A^{f}{(\frac{T}{T_{ref}})}^{n^{f}}exp(\frac{{-E}^{f}}{R_{g}T})$ |
|  |  |
| **Energy balance** | 𝜌𝐶𝑝𝜕𝑇𝜕𝑡+𝜌𝐶𝑝𝑢∙𝛻𝑇=𝛻∙(𝑘𝛻𝑇)+𝑄 |
|  | |
| **Mass transfer: Two-film theory** | $\frac{\partial\emptyset_{g}\rho_{g}}{\partial t}+\nabla\cdot\mathbf{N}_{\rho_{g}\emptyset_{g}}=-m_{gl}$ |
|  | $c^{*}=\frac{p+ p_{ref}}{H}$ |
|  | $N=k_{L}\left( c^{*}-c \right)$ |
|  | $m_{gl}=k\left( c^{*}-c \right)Ma$ |
|  | $\frac{\partial c}{\partial t}+\nabla\cdot\left( c\mathbf{u}_{l} \right)=\nabla\cdot\left( D\nabla c \right)+\frac{m_{gl}}{M}$ |
|  | |
| **Microbial growth** | $\Psi^{growth}=\frac{1}{t_{e}}\frac{n}{1+\frac{n^{2}}{n_{max}^{2}}}\left( \frac{c^{S}}{\alpha+c^{S}} \right)$ |
|  | $\Psi_{M-A-S}^{growth}= \Psi_{M-A-S,max}^{growth}\prod_{i} \left( \frac{c_{w}^{H_{2},{CO}_{2},C_{2}H_{4}O_{2},C_{6}H_{12}O_{6},{SO}_{4}}}{\alpha_{M-A-S, i}+ c_{w}^{H_{2},{CO}_{2},C_{2}H_{4}O_{2},C_{6}H_{12}O_{6,}{SO}_{4}}} \right)$ |


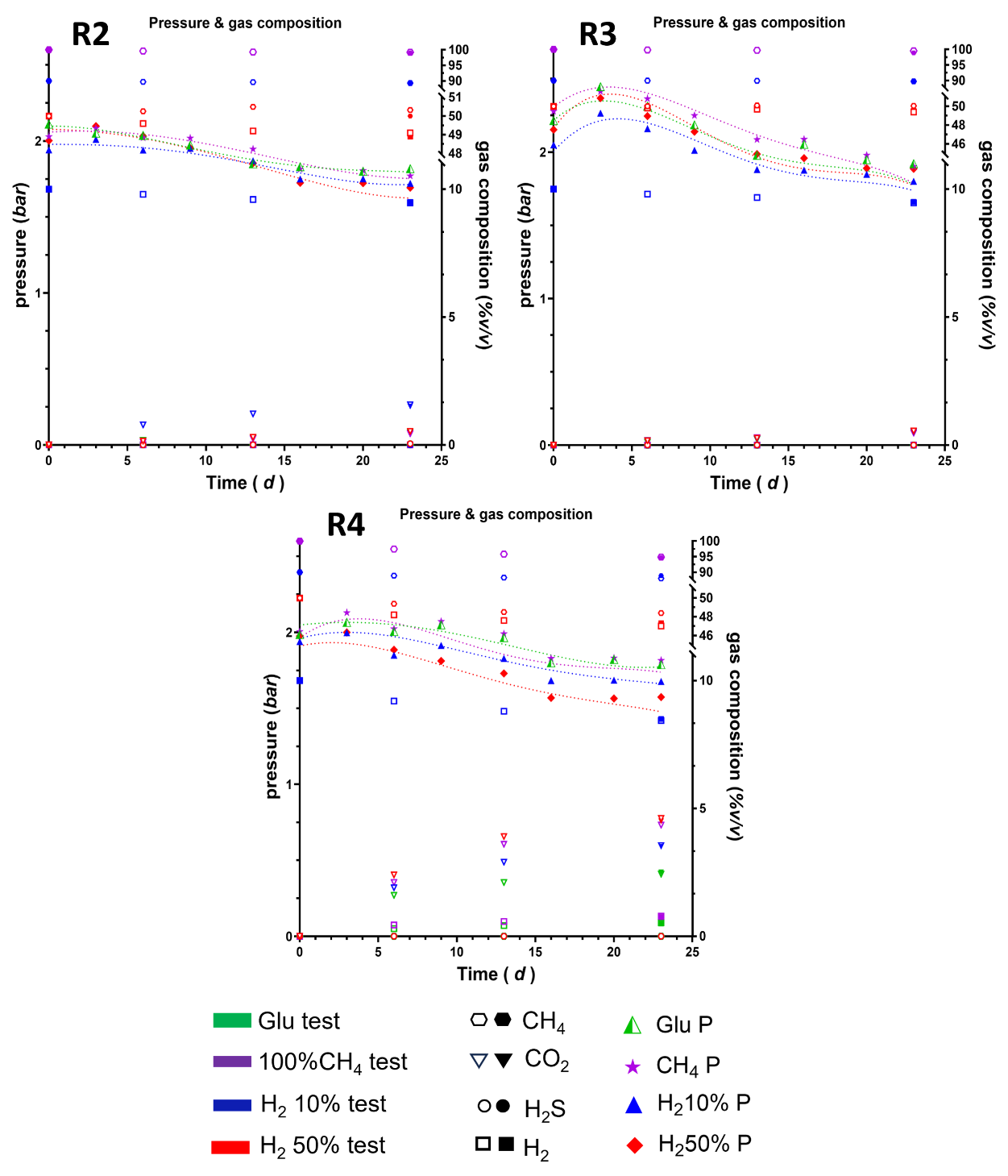


**Supplementary Figure 5 – S6.** Experimental (filled symbol) and simulated trends (dash lines for pressure, empty symbols for gas phase) of pressure and gas composition during batch cultivation of R2-R3-R4 formation waters under different CH_4_/H_2_ gas mixtures.

**
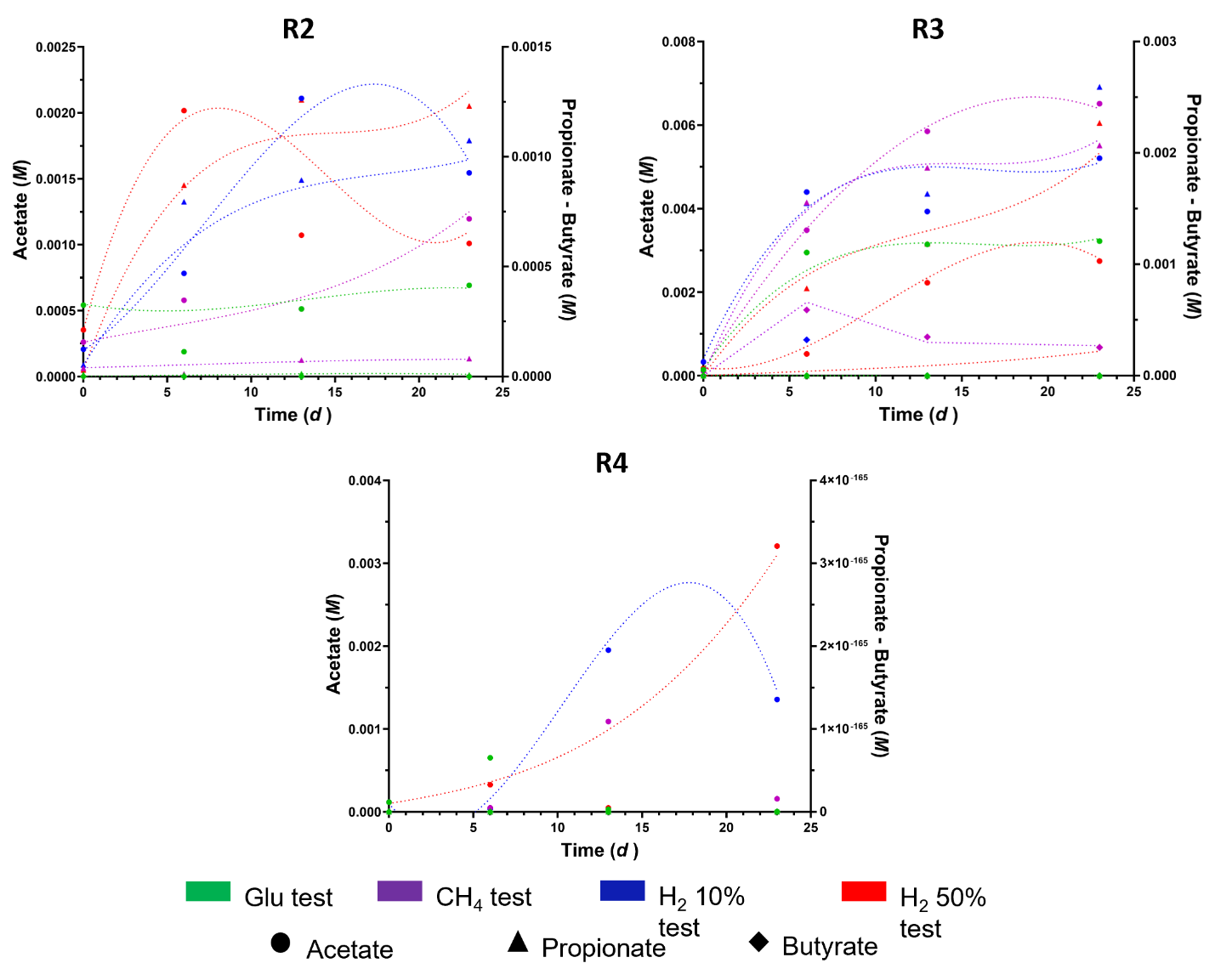
**

**Supplementary Figure 6 – S7.** Experimental (filled symbol) and simulated trends(dash lines) for VFA during batch cultivation of R2-R3-R4 formation waters under different CH_4_/H_2_ gas mixtures.

**
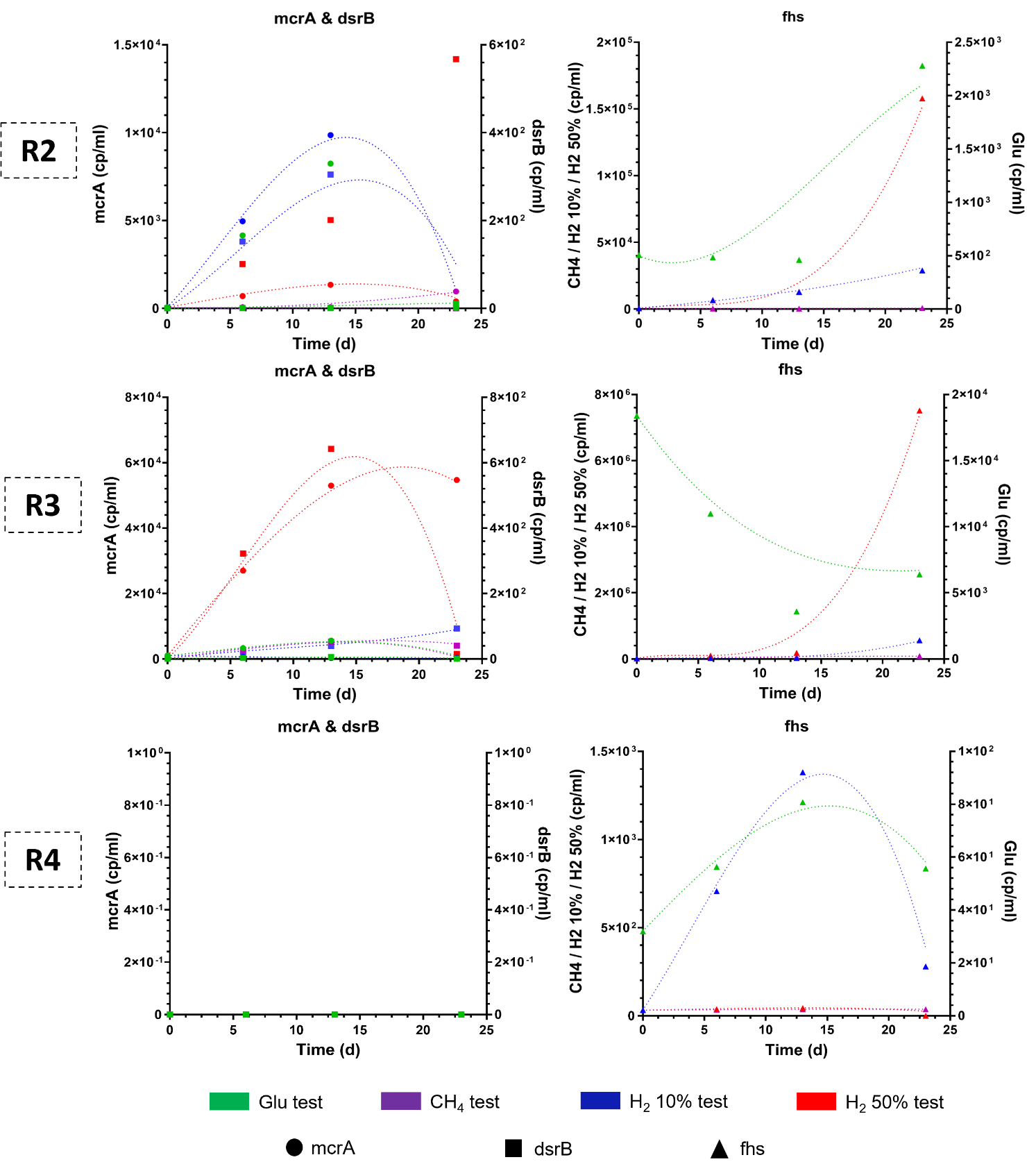
**

**Supplementary Figure 7 – S8.** Experimental (filled symbol) and simulated trends(dash lines) of copies/ml of mcrA, dsrB and fhs during batch cultivation of R2-R3-R4 formation waters under different CH_4_/H_2_ gas mixtures.

Table S9: Modelled gas consumption and production for the four reservoir object of the present study (continues nest page).

|  |  |  | **H2 - %v** | | **H2S - %v** | | **CO2 - %v** | | **CH4 - %v** | |
| --- | --- | --- | --- | --- | --- | --- | --- | --- | --- | --- |
|  |  | **Time** | *Exp* | *model* | *Exp* | *model* | *Exp* | *model* | *Exp* | *model* |
| **R1** | *Glu* | *Day 0* | 0 | 0 | 0 | 0 | 0 | 0 | 0 | 0 |
|  |  | *Day 6* |  | 1,2 |  | 2.9e-4 |  | 1,88 |  | 0 |
|  |  | *Day 13* |  | 1,07 |  | 0,005 |  | 3,27 |  | 0 |
|  |  | *Day 23* | 1 | 0,97 | 0,048 | 0,032 | 5,5 | 5,68 | 0 | 0 |
|  | *CH_4_* | *Day 0* | 0 | 0 | 0 | 0 | 0 | 0 | 100 | 100 |
|  |  | *Day 6* |  | 0,63 |  | 0 |  | 0,47 |  | 98,87 |
|  |  | *Day 13* |  | 0,59 |  | 0 |  | 1,87 |  | 97,48 |
|  |  | *Day 23* | 0,55 | 0,53 | 0 | 0 | 3,5 | 3,24 | 96,45 | 96,08 |
|  | *H_2_ 10%* | *Day 0* | 10 | 10 | 0 | 0 | 0 | 0 | 90 | 90 |
|  |  | *Day 6* |  | 9,52 |  | 0,00007 |  | 0,78 |  | 89,7 |
|  |  | *Day 13* |  | 8,99 |  | 0,000132 |  | 1,47 |  | 89,54 |
|  |  | *Day 23* | 8,5 | 8,34 | 0,048 | 0,014 | 2,5 | 2,18 | 88,5 | 88,48 |
|  | *H_2_ 50%* | *Day 0* | 50 | 50 | 0 | 0 | 0 | 0 | 49 | 50 |
|  |  | *Day 6* |  | 49,6 |  | 0,0004 |  | 0,5 |  | 49,86 |
|  |  | *Day 13* |  | 49,26 |  | 0,002 |  | 0,98 |  | 49,75 |
|  |  | *Day 23* | 48,5 | 48,7 | 0,003 | 0,0037 | 1,5 | 1,56 | 49 | 49,65 |
| **R2** | *Glu* | *Day 0* | 0 | 0 | 0 | 0 | 0 | 0 | 0 | 0 |
|  |  | *Day 6* |  | 0 |  | 0,00023 |  | 0,18 |  | 0 |
|  |  | *Day 13* |  | 0 |  | 0,004 |  | 0,26 |  | 0 |
|  |  | *Day 23* | 0 | 0 | 0,048 | 0,053 | 0,5 | 0,48 | 0 | 0 |
|  | *CH_4_* | *Day 0* | 0 | 0 | 0 | 0 | 0 | 0 | 100 | 100 |
|  |  | *Day 6* |  | 0 |  | 0 |  | 0,1 |  | 99,6 |
|  |  | *Day 13* |  | 0 |  | 0 |  | 0,24 |  | 99,3 |
|  |  | *Day 23* | 0 | 0 | 0 | 0 | 0,5 | 0,44 | 99 | 99,2 |
|  | *H_2_ 10%* | *Day 0* | 10 | 10 | 0 | 0 | 0 |  | 90 | 90 |
|  |  | *Day 6* |  | 9,8 |  | 0,0004 |  |  |  | 89,7 |
|  |  | *Day 13* |  | 9,6 |  | 0,00087 |  |  |  | 89,6 |
|  |  | *Day 23* | 9,5 | 9,47 | 0,003 | 0,0025 | 1,5 |  | 89,5 | 89,3 |
|  | *H_2_ 50%* | *Day 0* | 50 | 50 | 0 | 0 | 0 | 0 | 50 | 50 |
|  |  | *Day 6* |  | 49,6 |  | 0,00067 |  | 0,15 |  | 50,24933 |
|  |  | *Day 13* |  | 49,2 |  | 0,006 |  | 0,3 |  | 50,494 |
|  |  | *Day 23* | 48,9 | 49,1 | 0,048 | 0,046 | 0,5 | 0,53 | 50 | 50,324 |

Table S9: follows supplementary table 9

|  |  |  | **H2 - %v** | | **H2S - %v** | | **CO2 - %v** | | **CH4 - %v** | |
| --- | --- | --- | --- | --- | --- | --- | --- | --- | --- | --- |
|  |  | **Time** | *Exp* | *model* | *Exp* | *model* | *Exp* | *model* | *Exp* | *model* |
| **R3** | *Glu* | *Day 0* | 0 | 0 | 0 | 0 | 0 | 0 | 0 | 0 |
|  |  | *Day 6* |  | 0,3 |  | 0 |  | 1,6 |  | 0 |
|  |  | *Day 13* |  | 0,42 |  | 0 |  | 2,1 |  | 0 |
|  |  | *Day 23* | 0,5 | 0,53 | 0 | 0 | 2,5 | 2,42 | 0 | 0 |
|  | *CH_4_* | *Day 0* | 0 | 0 | 0 | 0 | 0 | 0 | 100 | 100 |
|  |  | *Day 6* |  | 0,44 |  | 0 |  | 2,1 |  | 97,46 |
|  |  | *Day 13* |  | 0,58 |  | 0 |  | 3,6 |  | 95,82 |
|  |  | *Day 23* | 0,75 | 0,788 | 0 | 0 | 4,5 | 4,35 | 94,7 | 94,862 |
|  | *H_2_ 10%* | *Day 0* | 10 | 10 | 0 | 0 | 0 | 0 | 90 | 90 |
|  |  | *Day 6* |  | 9,2 |  | 0,0004 |  | 1,9 |  | 88,8996 |
|  |  | *Day 13* |  | 8,8 |  | 0,00094 |  | 2,9 |  | 88,29906 |
|  |  | *Day 23* | 8,5 | 8,44 | 0,003 | 0,00263 | 3,5 | 3,55 | 88,9 | 88,00737 |
|  | *H_2_ 50%* | *Day 0* | 50 | 50 | 0 | 0 | 0 | 0 | 50 | 50 |
|  |  | *Day 6* |  | 48,2 |  | 0,00044 |  | 2,4 |  | 49,39956 |
|  |  | *Day 13* |  | 47,6 |  | 0,001 |  | 3,9 |  | 48,499 |
|  |  | *Day 23* | 47,3 | 47 | 0,003 | 0,0028 | 4,5 | 4,61 | 48,4 | 48,3872 |
| **R4** | *Glu* | *Day 0* | 0 | 0 | 0 | 0 | 0 | 0 | 0 | 0 |
|  |  | *Day 6* |  | 0 |  | 0,0001 |  | 0,11 |  | 0 |
|  |  | *Day 13* |  | 0 |  | 0,0013 |  | 0,23 |  | 0 |
|  |  | *Day 23* | 0 | 0 | 0,003 | 0,0035 | 0,5 | 0,54 | 0 | 0 |
|  | *CH_4_* | *Day 0* | 0 | 0 | 0 | 0 | 0 | 0 | 100 | 100 |
|  |  | *Day 6* |  | 0 |  | 0 |  | 0,16 |  | 99,6 |
|  |  | *Day 13* |  | 0 |  | 0 |  | 0,29 |  | 99,4 |
|  |  | *Day 23* | 0 | 0 | 0 | 0 | 0,5 | 0,47 | 98,9 | 99,1 |
|  | *H_2_ 10%* | *Day 0* | 10 | 10 | 0 | 0 | 0 | 0 | 90 | 90 |
|  |  | *Day 6* |  | 9,8 |  | 0 |  | 0,18 |  | 89,95 |
|  |  | *Day 13* |  | 9,67 |  | 0 |  | 0,25 |  | 89,9 |
|  |  | *Day 23* | 9,5 | 9,46 | 0 | 0 | 0,5 | 0,53 | 89,9 | 89,73 |
|  | *H_2_ 50%* | *Day 0* | 50 | 50 | 0 | 0 | 0 | 0 | 50 | 50 |
|  |  | *Day 6* |  | 49,82 |  | 0,0002 |  | 0,17 |  | 50,01 |
|  |  | *Day 13* |  | 49,7 |  | 0,0018 |  | 0,27 |  | 50,04 |
|  |  | *Day 23* | 49,5 | 49,4 | 0,003 | 0,0024 | 0,5 | 0,55 | 50 | 50,1 |

**Supplementary Table S10.** Simulated values (mols) of carbon uptake in CH_4_, CO_2_, Acetate, Propionate and Butyrate calculated by the model

|  |  |  |  |  |  |  |  |
| --- | --- | --- | --- | --- | --- | --- | --- |
|  |  | CH4 | CO2 | Acetate | Propionate | Butyrate | Tot |
| **R1** | **Glucose** |  | 0,0001865 | 0,0014614 | 0,0002685 | 0,0000000 | 0,0019164 |
|  | **100CH4** |  | 0,0001164 | 0,0005147 | 0,0001010 | 0,0000728 | 0,0008049 |
|  | **90CH4-10H2** | 3,65922E-06 | 0,0000790 | 0,0004085 | 0,0001986 | 0,0001001 | 0,0009544 |
|  | **50CH4-50H2** | 5,38439E-06 | 0,0000416 | 0,0003154 | 0,0001672 | 0,0000845 | 0,0006140 |
| **R2** | **Glucose** |  | 0,0000170 | 0,0000151 | 0,0000000 | 0,0000000 | 0,0000325 |
|  | **100CH4** |  | 0,0000165 | 0,0000936 | 0,0000064 | 0,0000000 | 0,0001165 |
|  | **90CH4-10H2** |  | 0,0000482 | 0,0001338 | 0,0001532 | 0,0000000 | 0,0003351 |
|  | **50CH4-50H2** |  | 0,0000158 | 0,0000656 | 0,0001802 | 0,0000000 | 0,0002616 |
| **R3** | **Glucose** |  | 0,00008334 | 0,00030772 | 0,00000000 | 0,00000000 | 0,00039106 |
|  | **100CH4** |  | 0,00015238 | 0,00064019 | 0,00031001 | 0,00005086 | 0,00115345 |
|  | **90CH4-10H2** | 0,00000543 | 0,00010947 | 0,00048772 | 0,00038889 | 0,00000000 | 0,00098608 |
|  | **50CH4-50H2** | 0,00000553 | 0,00013207 | 0,00025759 | 0,00034039 | 0,00005076 | 0,00076289 |
| **R4** | **Glucose** |  | 0,00001791 | 0,00001115 |  |  | 0,00002906 |
|  | **100CH4** |  | 0,00001791 | 0,00009740 |  |  | 0,00011512 |
|  | **90CH4-10H2** |  | 0,00001679 | 0,00012485 |  |  | 0,00014079 |
|  | **50CH4-50H2** |  | 0,00001758 | 0,00030920 |  |  | 0,00032678 |

|  |  | H2 uptake | | | |  |
| --- | --- | --- | --- | --- | --- | --- |
|  |  | Acetato | H2S | CH4 | Tot |  |
| **R1** | **Glucose** | 0,002922807 | 0 |  | 0,0029228 |  |
|  | **100CH4** | 0,001029491 | 0 |  | 0,0010295 |  |
|  | **90CH4-10H2** | 0,000816974 | 6,06602E-06 | 1,4637E-05 | 0,0008377 |  |
|  | **50CH4-50H2** | 0,000630827 | 3,325E-07 | 2,1538E-05 | 0,0006527 |  |
| **R2** | **Glucose** | 0,000030133 | 0 |  | 0,0000301 |  |
|  | **100CH4** | 0,000187155 | 0 |  | 0,0001872 |  |
|  | **90CH4-10H2** | 0,000267525 | 3,85281E-07 |  | 0,0002679 |  |
|  | **50CH4-50H2** | 0,000131246 | 6,06005E-06 |  | 0,0001373 |  |
| **R3** | **Glucose** | 0,00061544 | 0,00000000 |  | 0,00061544 |  |
|  | **100CH4** | 0,00128039 | 0,00000000 |  | 0,00128039 |  |
|  | **90CH4-10H2** | 0,00097545 | 0,00000038 | 0,00002170 | 0,00099753 |  |
|  | **50CH4-50H2** | 0,00051518 | 0,00000035 | 0,00002211 | 0,00053765 |  |
| **R4** | **Glucose** | 0,00002230 | 0,00000000 |  | 0,00002230 |  |
|  | **100CH4** | 0,00019480 | 0,00000000 |  | 0,00019480 |  |
|  | **90CH4-10H2** | 0,00024970 | 0,00000000 |  | 0,00024970 |  |
|  | **50CH4-50H2** | 0,00061840 | 0,00000042 |  | 0,00061882 |  |

**Supplementary Table S11.** Simulated values (mols) of hydrogen uptake in VFA, H_2_S, and CH_4_ as calculated by the model


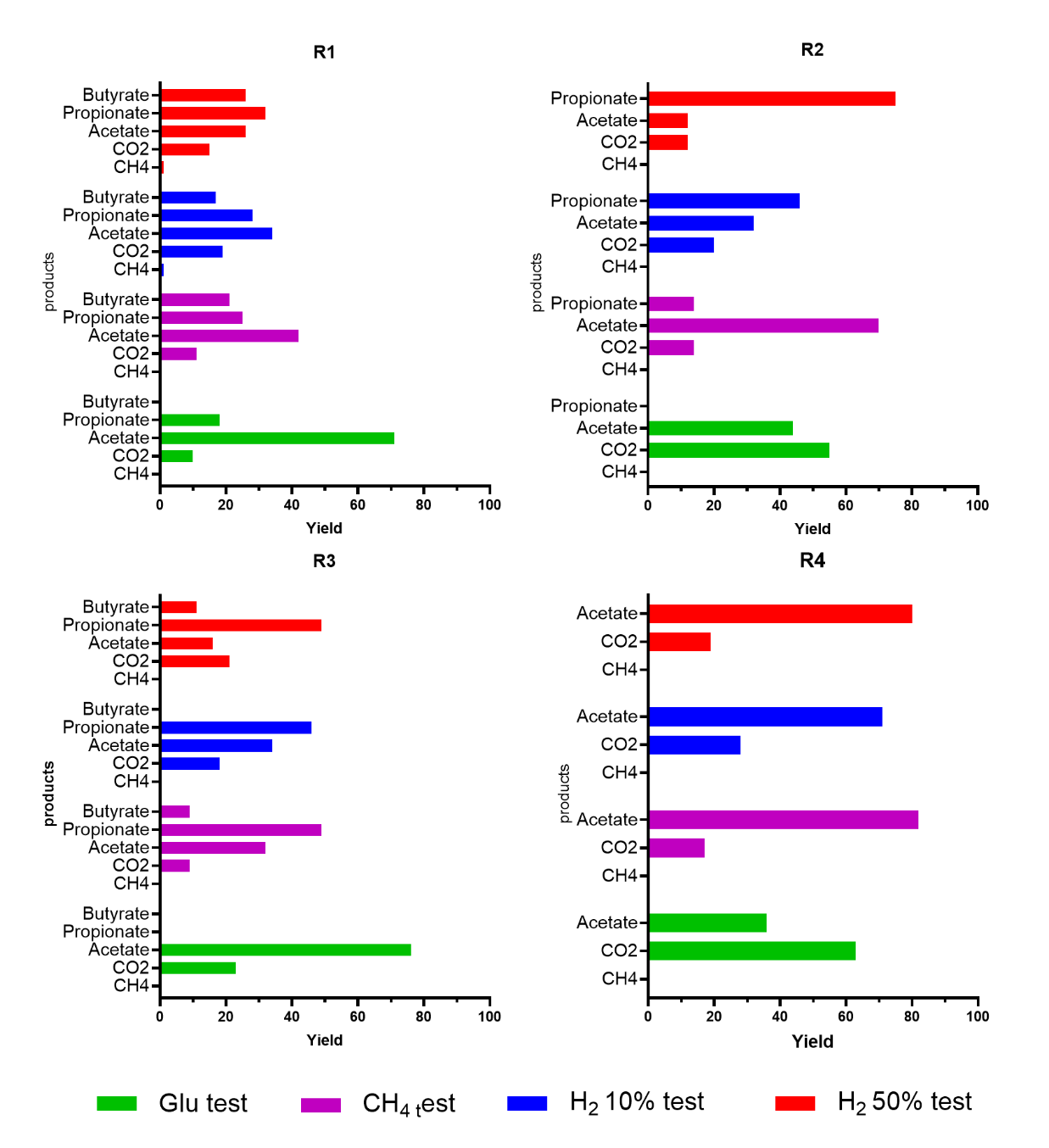


**Supplementary Figure 10 – S12.** Modelled values of carbon selectivity (Yield) after 90 days of simulated batch cultivation of R1-R2-R3-R4 formation waters under different CH_4_/H_2_ gas mixtures.
